# The AKR1C1–CYP1B1–cAMP signaling axis controls tumorigenicity and ferroptosis susceptibility of extrahepatic cholangiocarcinoma

**DOI:** 10.1038/s41418-024-01407-1

**Published:** 2024-10-30

**Authors:** Chang Liu, Cheng Zhang, Hongkun Wu, Zhibin Zhao, Zhenhua Wang, Xiaomin Zhang, Jieli Yang, Wenlong Yu, Zhexiong Lian, Minghui Gao, Lin Zhou

**Affiliations:** 1 Department of Laboratory Medicine, Shanghai Changzheng Hospital, Naval Medical University, Shanghai, China; 2 Institute of Aging & Tissue Regeneration, State Key Laboratory of Systems Medicine for Cancer, Ren-Ji Hospital, Shanghai Jiao Tong University School of Medicine (SJTU-SM), Shanghai, China; 3 Guangdong Provincial People’s Hospital (Guangdong Academy of Medical Sciences), Southern Medical University, Guangzhou, Guangdong China; 4 Department of Biliary Tract Surgery, Shanghai Eastern Hepatobiliary Surgery Hospital, Naval Medical University, Shanghai, China; 5 The HIT Center for Life Sciences, School of Life Science and Technology, Harbin Institute of Technology, Harbin, China

**Keywords:** Cancer genomics, Tumour biomarkers, Gene expression, Drug development

## Abstract

Extrahepatic cholangiocarcinoma (ECC), a highly malignant type of cancer with increasing incidence, has a poor prognosis due to limited treatment options. Based on genomic analysis of ECC patient samples, here we report that aldo-keto reductase family 1 member C1 (AKR1C1) is highly expressed in human ECC tissues and closely associated with ECC progression and poor prognosis. Intriguingly, we show that inducible AKR1C1 knockdown triggers ECC cells to undergo ferroptosis. Mechanistically, AKR1C1 degrades the protein stability of the cytochrome P450 family member CYP1B1, a newly discovered mediator of ferroptosis, via ubiquitin-proteasomal degradation. Additionally, AKR1C1 decreases CYP1B1 mRNA level through the transcriptional factor aryl-hydrocarbon receptor (AHR). Furthermore, the AKR1C1–CYP1B1 axis modulates ferroptosis in ECC cells via the cAMP–PKA signaling pathway. Finally, in a xenograft mouse model of ECC, AKR1C1 depletion sensitizes cancer cells to ferroptosis and synergizes with ferroptosis inducers to suppress tumor growth. Therefore, the AKR1C1–CYP1B1–cAMP signaling axis is a promising therapeutic target for ECC treatment, especially in combination with ferroptosis inducers.

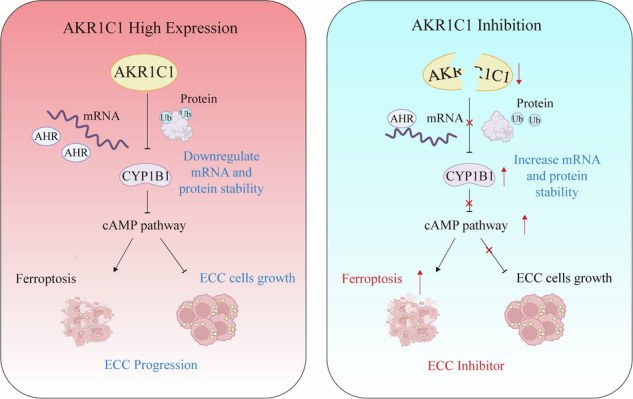

## Introduction

Cholangiocarcinoma (CCA) is a highly lethal malignant type of cancer arising from the epithelial cells of the bile, accounting for ~3% of the world’s annual gastrointestinal cancer and 15% of liver tumors, and the incidence of CCA has been gradually increasing in recent years [1, 2]. According to the anatomical distribution, CCA is classified into two main sub-types, that is, extrahepatic cholangiocarcinoma (ECC) and intrahepatic cholangiocarcinoma (ICC) [3]. As the major form of CCA, ECC accounts for ~80%–90% of CCA, and has a poor prognosis with no effective treatment except for complete resection. Moreover, ECC is difficult to be diagnosed at early stages due to its special anatomical region and asymptomatic state [4, 5]. To date, there is no drug that can cure ECC, and the treatment options are mainly limited to surgery, chemotherapy, and radiation therapy [6–8]. Further, genomic information of ECC in public domains such as The Cancer Genome Atlas Program (TCGA) database is scarce. Therefore, it remains an urgent and unmet clinical need for further exploring the pathogenic mechanism of ECC to identify new drivers and therapeutic targets to improve clinical management of patients with ECC.

Aldo-keto reductase family 1 member C1 (AKR1C1), a member of the aldo-keto reductase (AKR) family, catalyzes NADPH-dependent reduction, and thus plays essential roles in the metabolisms of steroid hormones, prostaglandins, and polycyclic aromatic hydrocarbons [9–11]. Some studies have implicated that AKR1C1 promotes cell proliferation, metastasis, and chemotherapy- resistance in multiple cancers. AKR1C1 is also reported to be upregulated in various cancers such as lung, breast, gastric, prostate, and cervical cancers [12–17]. Recently, AKR1C1 has been implicated in the regulation of ferroptosis [18–20], a form of regulated cell death characterized by iron and lipid reactive oxygen species (ROS) accumulation [21–24]. Although the physiological function of ferroptotic cell death remains elusive, growing lines of evidence suggest that ferroptosis dysfunction is highly related to various human diseases, including tumorigenesis [25, 26]. Inducing ferroptosis has emerged as an attractive strategy for managing various types of tumors [27–32].

Importantly, AKR1C1 is suggested to be a potential therapeutic target to promote cancer cell death under various contexts. For example, upregulated AKR1C1 results in ferroptotic resistance in melanoma and small cell lung carcinoma [18], and AKR1C1 inhibitors potentiate ferroptosis in colon cancer [33]. However, it remains to be greatly elusive how AKR1C1 mediates ferroptosis.

Herein, we demonstrate that AKR1C1 negatively impacts both mRNA and protein stability of the cytochrome P450 family member CYP1B1 to suppress ferroptosis, and that AKR1C1 depletion can promote ECC ferroptosis in both cells and xenograft models. Collectively, AKR1C1 is a promising therapeutic target for ECC treatment through sensitizing ECC tumor tissue to ferroptotic cell death.

## Results

### AKR1C1 is highly expressed in ECC

**Fig. 1:**
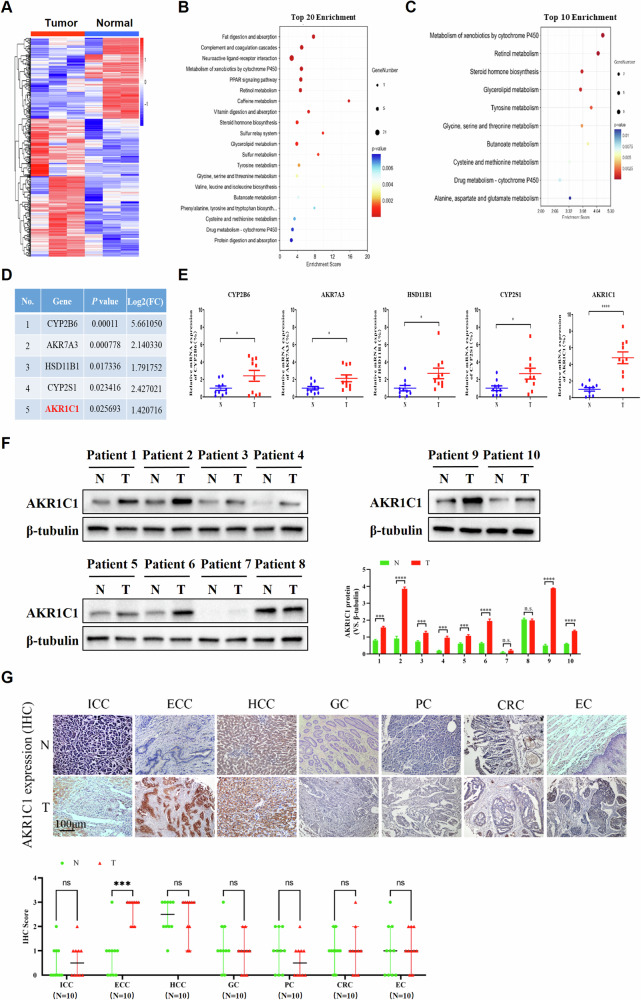
AKR1C1 is highly expressed in ECC. **A** RNA-seq heatmap showing differentially expressed genes (*P* value < 0.05 and |log_2_ fold-change| > 1) detected in three pairs of ECC patient samples versus adjacent non-tumor tissues (*n* = 3). **B** The KEGG analyses were performed and the top 20 enriched pathways among differentially expressed genes in (**A**) were shown by the bubble plot. **C** Among pathways in (**B**), 10 pathways related to metabolism were shown by the bar plot, and cytochrome P450-mediated metabolism of xenobiotics was identified among them (*P* value = 1.19E − 05). **D** A list of the top 5 genes with the most significant differences in the cytochrome P450 metabolic pathway identified, including AKR1C1. **E** qRT-PCR analysis of the five genes with the highest differential expression in the cytochrome P450 metabolic pathway in clinical samples from ten cases of ECC and matched adjacent tissues (*n* = 10). Glyceraldehyde-3-phosphate dehydrogenase (GAPDH) expression was used as an internal control for normalization. *N* normal tissues, T tumor tissues. **F** Quantification of the IB analysis confirmed the elevated expression of AKR1C1 protein in human ECC specimens compared with precancerous tissues (*n* = 10). Beta tubulin was used as the loading control. **G** Representative IHC staining results of AKR1C1 and the staining scores in another 70 pairs of clinical tumor samples from ICC (*n* = 10), ECC (*n* = 10), hepatocellular carcinoma (HCC) (*n* = 10), gastric cancer (GC) (*n* = 10), pancreatic cancer (PC) (*n* = 10), colorectal cancer (CRC) (*n* = 10), esophageal cancer (EC) (*n* = 10) and the corresponding normal tissue. **E**–**G** Data are presented as the mean ± standard deviation from three independent experiments, n.s. > 0.05, *P < 0.05, ***P < 0.001, ****P < 0.0001.

To identify candidate genes that might contribute to ECC pathogenesis, we performed high-throughput RNA sequencing (RNA-seq) in three pairs of human primary ECC tissues and adjacent non-tumor tissues. A total of 409 differentially expressed genes, including 258 upregulated genes and 151 downregulated genes, were identified (*P* value < 0.05 and |log_2_ fold-change| > 1) in ECC primary tumor tissues compared with the corresponding normal tissues (Fig. 1A). The Kyoto Encyclopedia of Genes and Genomes (KEGG) analyses revealed the top 20 significantly enriched pathways (Fig. 1B), of which, 10 pathways were associated with metabolism. As shown in Fig. 1C, the most significantly associated metabolic pathway was metabolism of xenobiotics by cytochrome P450 (*P* value = 1.19E − 05). Notable, a set of genes involved in cytochrome P450-mediated xenobiotics metabolism were identified. The top five genes were individually validated by detecting their mRNA expression in clinical tumor tissues from ten cases of ECC patients and matched adjacent tissues. The results revealed that AKR1C1 was the most differentially expressed genes in ECC tissues (Fig. 1D, E). As AKR1C1 is recently shown to be involved in human cholangiocarcinoma [34], we chose it for further investigation.

To analyze AKR1C1 protein expression in these ten pairs of ECC patient samples by immunoblotting (IB) assay, we found that there was elevated expression of AKR1C1 in the majority of human ECC specimens (8 cases/10 cases, 80%), compared with matched non-tumor tissues (Fig. 1F). Applying immunohistochemical (IHC) staining to examine AKR1C1 expression in another 70 pairs of clinical tumor samples from ICC, ECC, hepatocellular carcinoma (HCC), gastric cancer (GC), pancreatic cancer (PC), colorectal cancer (CRC), esophageal cancer (EC) and the corresponding normal tissues, we also revealed that the ECC tissues had markedly stronger AKR1C1 immunostaining than other types of tumor samples, including ICC (Fig. 1G). In addition, qRT-PCR analysis also showed that the extrahepatic CCA cell line QBC939 had a higher expression of AKR1C1 than cell lines of other tumor types (Supplementary Fig. 1). Collectively, these analyses suggest that AKR1C1 may play a crucial role in the pathogenesis of ECC.

### Overexpression of AKR1C1 is correlated with ECC progression and poor prognosis

**Fig. 2:**
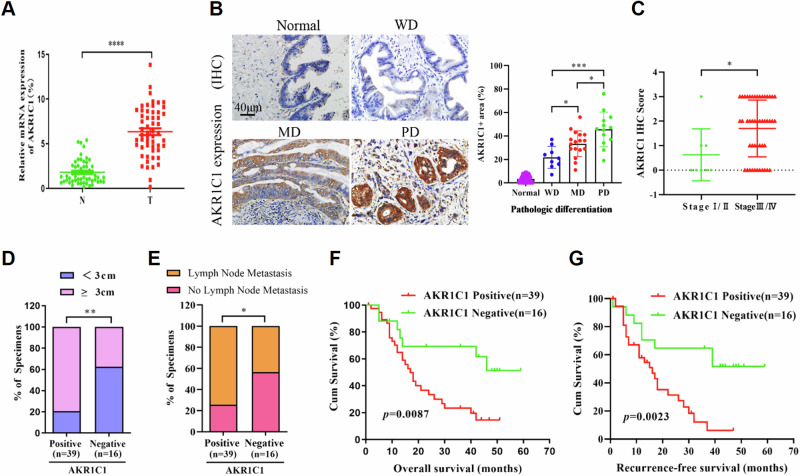
Overexpression of AKR1C1 is correlated with ECC progression and poor prognosis. **A** qRT-PCR analysis of AKR1C1 expression in 55 pairs of human primary ECC tissues and adjacent non-tumor tissues. GAPDH expression was used as an internal control for normalization. N normal tissues, T tumor tissues. **B** Representative IHC staining of AKR1C1 in normal extrahepatic bile duct tissue and different histologic grade of human ECC specimens (*n* = 55), including well differentiated, moderately differentiated, and poorly differentiated. Statistical analysis results are shown on the right, based on quantification of the percentage of AKR1C1^+^ area in each tissue section. WD well differentiation, MD moderate differentiation, PD poor differentiation. **C** Dot distribution graph of AKR1C1 IHC staining scores was shown in 55 ECC patients of different clinical stages. **D** Correlation between AKR1C1 protein expression and tumor diameter (*n* = 55). **E** Correlation between AKR1C1 protein expression and lymph node metastasis (*n* = 55). **F** Kaplan–Meier analysis of overall survival and **G** recurrence-free survival based on AKR1C1 expression in 55 ECC patients. Data are presented as the mean ± standard deviation from three independent experiments, n.s. > 0.05, *P < 0.05, **P < 0.01, ***P < 0.001, ****P < 0.0001.

To further corroborate the clinical significance of AKR1C1 overexpression in ECC progression, the tumor tissues and matched adjacent normal tissues from 55 different ECC patients were harvested for qRT-PCR. Their clinical and pathological characteristics were summarized in Table 1. The results showed that AKR1C1 mRNA level was significantly upregulated in ECC tissues compared to that in the adjacent non-tumor tissues (Fig. 2A).

**Table 1:** Clinical and pathological characteristics of 55 diagnosed ECC patients.

Clinical features	No. of cases	AKR1C1 positive cases (%)	*P* value
Gender			0.262
Male	35	23 (65.7)	
Female	20	16 (80.0)	
Age (years)			0.090
≤60	27	22 (81.5)	
>60	28	17 (60.7)	
TNM stage			0.0038**
I/II	8	3 (37.5)	
III/IV	47	36 (76.6)	
Tumor size			0.003**
<3 cm	18	8 (44.4)	
≥3 cm	37	31 (83.8)	
Histologic grade			0.007**
Well	20	9 (45.0)	
Moderate	21	17 (81.0)	
Poor	14	13 (92.9)	
Lymph node metastasis			0.030*
No	19	10 (52.6)	
Yes	36	29 (80.6)	

*P values < 0.05 were considered statistically significant.

**P < 0.01.

We subsequently assessed the expression of AKR1C1 protein by IHC staining in these ECC patient samples. Our results indicated that AKR1C1 was positively expressed in 70.9% (39/55) of the ECC tumor samples compared with matched adjacent tissues (Table 1), and AKR1C1 protein expression was positively corrected with histologic grade (*P* = 0.007, Fig. 2B) and Tumor-Node-Metastasis (TNM) stage (*P* = 0.0038, Fig. 2C) of tumors, whereas it had no significant correlation with sex or age. The proportion of AKR1C1-positive patients tended toward larger tumor size (≥3 cm) (*P* = 0.003) and higher risk of lymph node metastasis (*P* = 0.03) (Table 1 and Fig. 2D, E).

Furthermore, the Kaplan–Meier survival analysis revealed that positive expression of AKR1C1 was significantly associated with poor overall survival (OS, *P* = 0.0087) and short recurrence-free survival (RFS, *P* = 0.0023) in this cohort of 55 patients with ECC (Fig. 2F, G). Taken together, these findings suggest that AKR1C1 overexpression is closely associated with ECC tumor progression and may be potentially used as a diagnostic and prognostic marker for ECC.

### AKR1C1 knockdown inhibits ECC tumor growth in vitro and in vivo

**Fig. 3:**
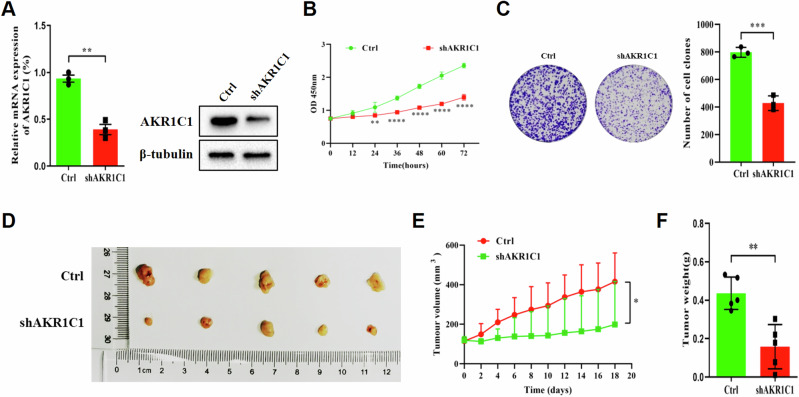
AKR1C1 knockdown inhibits ECC tumor growth in vitro and in vivo. **A**–**C** QBC939 cells were infected with lentiviral particles containing Dox-inducible shRNA against AKR1C1 (shAKR1C1 QBC939 cells), followed with or without Dox (5 µg/ml). **A** Expression of AKR1C1 was assayed by qRT-PCR (left) and western blot (right). **B** Cell proliferation was tested by CCK-8 assay. **C** Observe the colony forming ability of the cells and take pictures (left) after 14 days. Statistical chart of cell counts (right). **D**–**F** QBC939 cells harboring Dox-inducible shAKR1C1 were subcutaneously injected into nude mice and treated with normal feeding or DOX feeding (*n* = 6/group). **D** Images of resected tumors from each group were taken and **E** tumor volumes of each treatment group were measured every 2 days. Data are presented as the mean ± standard, *P < 0.05. **F** Tumor weights were measured at day 18. **A**–**C** Data are presented as the mean ± standard deviation from three independent experiments, **P < 0.01, ***P < 0.001, ****P < 0.0001.

To investigate the functional role of AKR1C1 in ECC progression, we downregulated AKR1C1 expression in QBC939 cells using a doxycycline (Dox)-inducible system. Dox-induced AKR1C1 short hairpin RNA (shRNA) effectively inhibited AKR1C1 expression in QBC939 cells (Fig. 3A) and reduced cell proliferation and colony formation (Fig. 3B, C).

We also employed a QBC939 cells-derived xenograft mouse model to investigate whether AKR1C1 expression is essential for tumor growth in vivo. As shown in Fig. 3D–F, QBC939 cells with Dox-inducible depletion of AKR1C1 significantly impaired tumor growth, as indicated by tumor size, tumor volume, and tumor weight. Overall, these results indicate that AKR1C1 plays a crucial role in promoting ECC tumor formation.

### AKR1C1 inhibition triggers ferroptosis in ECC cells

**Fig. 4:**
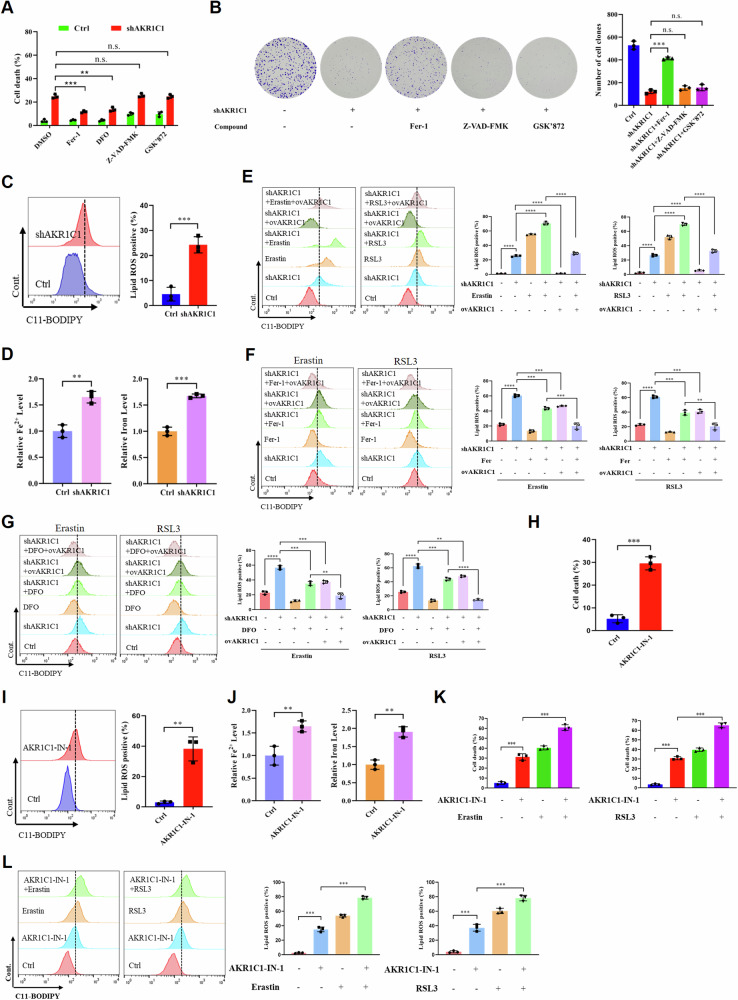
AKR1C1 inhibition triggers ferroptosis in ECC cells. **A** QBC939 cells containing Dox-inducible shAKR1C1 in the presence or absence of Dox (5 µg/ml) were treated with Fer-1 (2 µM), DFO (5 µM), Z-VAD-FMK (2 µM), or GSK′872 (2 µM) for 22 h. Cell death was quantified by SYTOX Green staining. **B** shAKR1C1 QBC939 cells in the presence or absence of Dox (5 µg/ml) were treated with Fer-1 (2 µM), Z-VAD-FMK (2 µM), or GSK′872 (2 μM) for 7 days. Observe the colony forming ability of the cells and take pictures (left). Statistical chart of cell counts (right). **C** Lipid ROS measurement in QBC939 cells containing Dox-inducible shAKR1C1 treated with or without Dox (5 µg/ml) for 48 h. **D** Levels of Fe^2+^ (left) and total iron (right) were analyzed in shAKR1C1 QBC939 cells in the presence or absence of Dox (5 µg/ml). **E** QBC939 cells containing Dox-inducible shAKR1C1 were cultured in the presence of Dox (5 µg/ml) and treated with Erastin (30 μM), RSL3 (2 µM) or transfected with wild-typed pcDNA-AKR1C1 vector. Lipid ROS was assessed at 9 h after treatment. **F** QBC939 cells were prepared as described in (**E**) and treated with or without Fer-1 (2 μM). Lipid ROS was measured at 9 h. **G** QBC939 cells were prepared as described in (**E**) and treated with or without DFO (5 µM). Lipid ROS was assessed at 9 h after treatment. **H**–**J** QBC939 cells were supplemented with the AKR1C1 selective inhibitor AKR1C1-IN-1 (10 nM). **H** Cell death was quantified by SYTOX Green staining. **I** Lipid ROS measurement in QBC939 cells. **J** Levels of Fe^2+^ (left) and total iron (right). **K**, **L** QBC939 cells were prepared as described in (**H**–**J**) and treated with Erastin (30 µM) or with RSL3 (2 µM). **K** Cell death was quantified by SYTOX Green staining. **L** Lipid ROS was assessed at 9 h after treatment. All the data are presented as the mean ± standard deviation from three independent experiments, n.s. > 0.05, **P < 0.01, ***P < 0.001, ****P < 0.0001.

Since AKR1C1 was previously demonstrated to be highly related to ferroptosis in some cancer cells [29, 30], we sought to determine whether AKR1C1 depletion could affect ferroptosis in ECC cells. To this end, we examined the impact of the ferroptosis inhibitor ferrostain-1 (Fer-1) or iron chelator deferoxamine (DFO), the apoptosis inhibitor Z-VAD-FMK, and the necroptosis inhibitor GSK′872, on the clonogenic survival of AKR1C1-depleted ECC cells. We found that Dox-induced AKR1C1 depletion markedly enhanced the cell death of ECC cells, importantly, the death of AKR1C1-depleted cells was significantly rescued only by treatment with Fer-1 and DFO, suggesting that inducible AKR1C1 knockdown may attenuate cell growth, at least partly, by inducing ferroptosis (Fig. 4A, B). To confirm this finding, we assessed intracellular iron and lipid ROS levels in AKR1C1-depleted ECC cells. As expected, AKR1C1 depletion increased intracellular lipid ROS, total iron, and Fe^2+^ levels in ECC cells (Fig. 4C, D).

To further probe the influence of AKR1C1 depletion on ferroptosis in ECC cells, we assessed sensitivity of the cells to RSL3 and erastin, two canonical ferroptosis inducers that act at different steps in the ferroptosis cascade. As shown in Fig. 4E–G, combining inducible AKR1C1 knockdown with either erastin or RSL3 treatment resulted in a much stronger effect on accumulation of lipid ROS in ECC cells than erastin or RSL3 treatment alone. As expected, these increase in ROS production could be rescued by co-incubation with Fer-1 and DFO. Importantly, overexpression of the wildtype AKR1C1 could offset AKR1C1 knockdown-mediated ferroptosis, confirming the effect of the inducible shRNA is specifically through knocking down AKR1C1.

We next explored whether blockade of AKR1C1 enzymatic activity could also affect ferroptosis in ECC cells, using a selective inhibitor of AKR1C1 (AKR1C1-IN-1). Indeed, similar ferroptosis-inducing effects were observed in ECC cells treated with AKR1C1-IN-1 in comparison with inducible AKR1C1 knockdown, including lipid ROS accumulation, increased intracellular iron levels, and more cell death. Moreover, the effect of erastin or RSL3 triggered ferroptosis was also dramatically enhanced by AKR1C1-IN-1 treatment (Fig. 4H–L). Taken together, AKR1C1 depletion induces ferroptosis and potentiates ferroptosis triggered by its canonical induces in ECC cells.

### AKR1C1 interacts directly with CYP1B1

To investigate how AKR1C1 exerts its functions in ferroptosis modulation in ECC cells, we performed co-immunoprecipitation (Co-IP) followed by liquid chromatography coupled with tandem mass spectrometry (LC–MS/MS) to identify potential binding proteins of AKR1C1. Given that AKR1C1 was associated with cytochrome P450-mediated xenobiotics metabolism pathway as shown in our RNA-seq analysis (Fig. 1C, D), we focused on one of the identified candidate binding proteins, cytochrome P450 1B1 (CYP1B1) (Supplementary Fig. 2), which is a member of the cytochrome P450 (CYP) enzyme family and likely to be involved in ferroptosis [35]. Further, several members of the CYP superfamily have been shown to play critical roles in ferroptosis. For example, cytochrome P450 oxidoreductase (CYPOR), NADH-cytochrome b5 reductase 1 (CYB5R1), cytochrome P450 cyclooxygenase 2J2 (CYP2J2), and cytochrome P450-2E1 (CYP2E1) were recently proved to be “executioners” for ferroptosis [36–41].

**Fig. 5:**
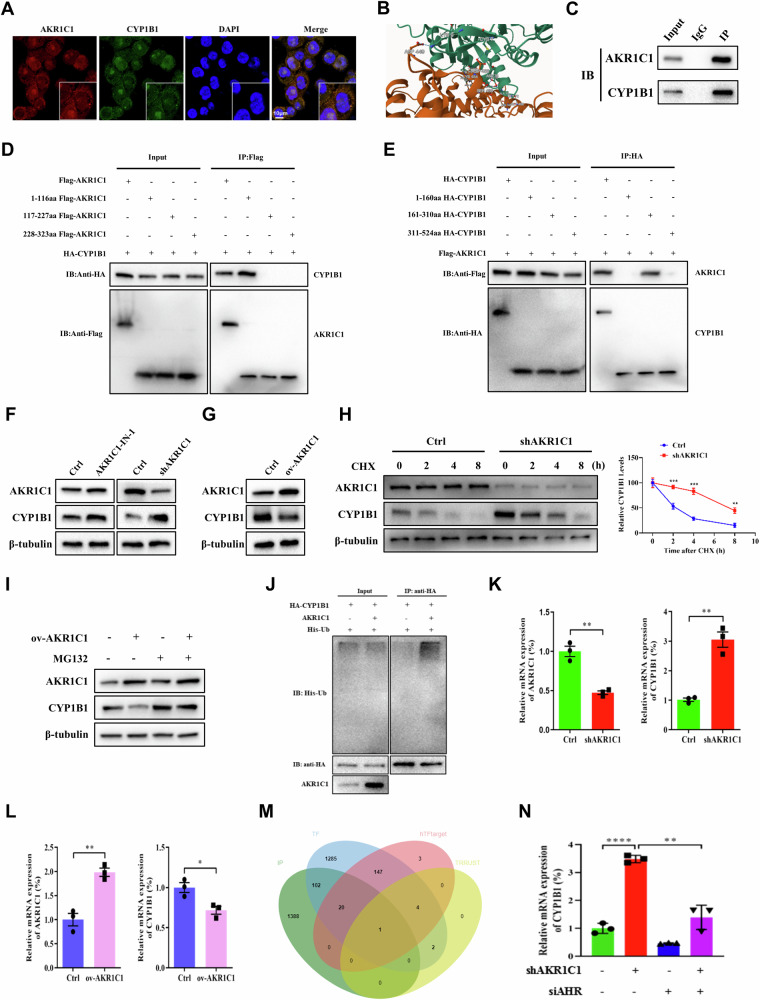
AKR1C1 directly binds to and downregulates both the mRNA and protein stability of CYP1B1. **A** The intracellular localization of AKR1C1 and CYP1B1 was confirmed by immunofluorescence assay. **B** AKR1C1 was globally docked with CYP1B1 protein using MEGADOCK, a protein–protein interaction prediction system. Orange indicates AKR1C1 and green indicates CYP1B1. **C** QBC939 cell lysates were prepared for Co-IP (co-immunoprecipitation). Immunoblot analysis was performed on the co-immunoprecipitated material using antibodies against AKR1C1 and CYP1B1. **D** Transfection of the HA-tagged CYP1B1 or Flag-tagged AKR1C1 and its serial domain truncation mutants together into 293T cells. The cell lysates were subjected to immunoprecipitation with Flag antibody, followed by western blotting to detected coprecipitation phenomena. **E** Transfection of Flag-tagged AKR1C1 or HA-tagged CYP1B1 and its serial domain truncation mutants together into 293T cells. The cell lysates were subjected to immunoprecipitation with HA antibody, followed by western blotting to detected coprecipitation phenomena. **F** QBC939 cells containing Dox-induced shAKR1C1 were treated with or without Dox (5 µg/ml). Alternatively, the AKR1C1 selective inhibitor AKR1C1-IN-1 (10 nM) was added to the cells. Western blotting was performed to detect the expression of AKR1C1 and CYP1B1 in QBC939 cells. **G** QBC939 cells were transfected with a plasmid overexpressing AKR1C1. After 48 h, the expression of AKR1C1 and CYP1B1 in QBC939 cells was assessed by western blotting. **H** QBC939 cells with or without inducible AKR1C1 knockdown were treated with 50 µg/ml CHX for 2, 4, and 8 h, as indicated. The expression of CYP1B1 was detected by western blot analysis (left) and quantitatively analyzed using ImageJ software (right). **I** QBC939 cells containing Dox-induced shAKR1C1 in the presence or absence of Dox (5 µg/ml) and treated with 10 µM MG132 for 6 h before western blotting analysis of the indicated proteins. **J** In vivo ubiquitination assays were performed in 293T cells transfected with His-Ub plasmid, HA-CYP1B1 plasmid or AKR1C1 plasmid, followed by western blot analysis with anti-HA and anti-His antibodies. **K** QBC939 cells were infected with lentiviral particles containing Dox-inducible shRNA against AKR1C1, followed with or without Dox (5 µg/ml). Expression of AKR1C1 and CYP1B1 was assayed by qRT-PCR. **L** AKR1C1 overexpression plasmid was transfected in QBC939 cells. qRT-PCR was used to detect the expression of AKR1C1 and CYP1B1 in the cells after 48 h. **M** A Venn diagram of IP binding proteins, transcription factor datasets, hTFtarget and TRRUST databases. **N** AKR1C1 and AHR were downregulated simultaneously or separately in QBC939 cells. qRT-PCR was performed to detect the expression of CYP1B1. **C**–**L** and **N** The data are presented as the mean ± standard deviation from three independent experiments, *P < 0.05, **P < 0.01, ***P < 0.001, ****P < 0.0001.

We confirmed AKR1C1–CYP1B1 interaction by the following experiments. First, immunofluorescence staining showed that AKR1C1 and CYP1B1 were co-localization both in the nucleus and cellular matrix in QBC939 cells (Fig. 5A). Second, both MEGADOCK protein interaction prediction analysis and co-immunoprecipitation assays validated their interaction (Fig. 5B, C). To identify essential domains for the interaction between AKR1C1 and CYP1B1, we expressed wildtype FLAG-tagged AKR1C1 or its serial domain truncation mutants, and wildtype HA-tagged CYP1B1 or its different truncation mutants in 293T cells. Then, Co-IP assays were performed using anti-HA or anti-FLAG antibodies, respectively. The results showed that the fragment of AKR1C1 containing the first 160 residues and that of CYP1B1 containing amino acid 161–310 residues were responsible for the interaction between AKR1C1 and CYP1B1 (Fig. 5D, E). Altogether, these data reveal that AKR1C1 directly binds to the cytochrome P450 family member CYP1B1, which is likely to be a mediator of ferroptosis.

### AKR1C1 negatively impacts both protein stability and mRNA level of CYP1B1

To determine whether AKR1C1 functions as a regulator of CYP1B1, we first examined the protein expression level of CYP1B1 by Western blotting in ECC cells with AKR1C1 blockade using AKR1C1 selective inhibitor AKR1C1-IN-1, knockdown AKR1C1 using Dox-induced AKR1C1 shRNA, and AKR1C1 overexpressed by Dox-induced AKR1C1 overexpression. In either AKR1C1-IN-1-treated or AKR1C1-depleted cells, we observed a markedly accumulation of CYP1B1 in protein level, as compared to controls. In contrast, inducible AKR1C1 overexpression significantly decreased the CYP1B1 protein level in ECC cells, suggesting that AKR1C1 downregulates CYP1B1 protein expression (Fig. 5F, G).

To investigate how AKR1C1 impacts CYP1B1 protein expression, a protein synthesis inhibitor cycloheximide (CHX) pulse-chase experiment was performed. In the presence of CHX, CYP1B1 protein level was remarkably decreased for different time points, and inducible AKR1C1 knockdown significantly alleviated the degradation rate of CYP1B1 protein (Fig. 5H).

Additionally, treatment with MG132, a proteasome inhibitor, slowed down CYP1B1 protein degradation upon AKR1C1 overexpression (Fig. 5I), indicating that AKR1C1 mediates proteasomal degradation of CYP1B1. Indeed, CYP1B1 was efficiently ubiquitylated when AKR1C1 was overexpressed (Fig. 5J). These findings indicate that AKR1C1 degrades CYP1B1 protein in ubiquitin-proteasomal degradation-dependent manner.

Intriguingly, AKR1C1 can also affect CYP1B1 transcription, as depletion of AKR1C1 significantly increased the mRNA level of CYP1B1, and conversely overexpressing AKR1C1 decreased the CYP1B1 mRNA level (Fig. 5K, L). Therefore, we hypothesize that AKR1C1 downregulates the mRNA of CYP1B1 through the transcription factor. The transcription factor prediction databases hTFtarget (https://guolab.wchscu.cn/hTFtarget/#!/) and TRRUST (https://www.grnpedia.org/trrust/) were utilized to predict the potential transcription factors for CYP1B1. We reasoned that the AKR1C1-regulated transcription factor for CYP1B1 might be a binding partner of AKR1C1. With the potential AKR1C1 binding proteins we identified, there is one transcription factor predicted to drive CYP1B1 transcription, named aryl-hydrocarbon receptor (AHR) (Fig. 5M). As shown in Fig. 5N, silencing AHR with siRNA significantly counteracted the accumulation of CYP1B1 mRNA level in AKR1C1-depleted ECC cells, indicating that AKR1C1 is capable of decreasing CYP1B1 mRNA level through AHR, a transcriptional factor. Collectively, these results demonstrate that AKR1C1 negatively impacts both mRNA and protein stability of the cytochrome P450 family member CYP1B1.

### The AKR1C1–CYP1B1 axis modulates ferroptosis via the cAMP–PKA signaling pathway in ECC cells

**Fig. 6:**
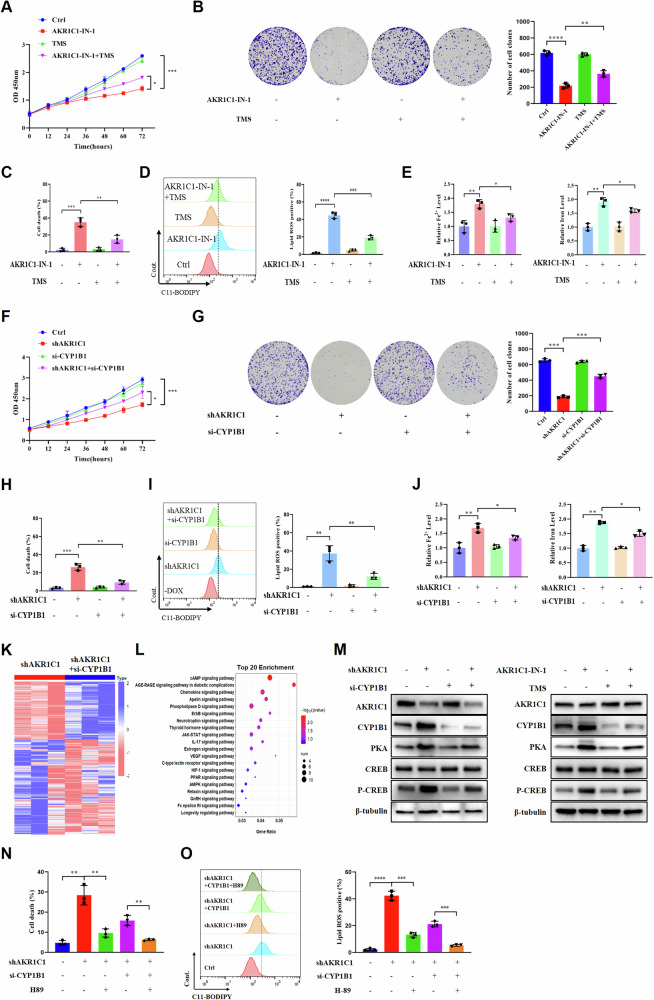
The AKR1C1–CYP1B1 axis modulates ferroptosis via the cAMP–PKA signaling pathway in ECC cells. **A**–**E** The AKR1C1 selective inhibitor AKR1C1-IN-1 (10 nM) was added to QBC939 cells with or without CYP1B1 inhibitor TMS (3 nM). **A** Cell proliferation was detected using CCK-8 and **B** the colony forming ability of the cells was observed. Flow cytometry to detect cell death (**C**) and intracellular lipid ROS (**D**). **E** Levels of Fe^2+^ (left) and total iron (right). **F**–**J** Downregulation of AKR1C1 expression in QBC939 cells with or without small interfering RNA for CYP1B1. **F** Cell proliferation was detected using CCK-8 and **G** the colony forming ability of the cells was observed. Flow cytometry to detect cell death (**H**) and intracellular lipid ROS (**I**). **J** Levels of Fe^2+^ (left) and total iron (right). **K** RNAs derived from QBC939 cells containing Dox-inducible shAKR1C1 following 48 h treatment with Dox (5 µg/ml) and with small interfering RNA for CYP1B1 were extracted and subjected to RNA sequencing. Heatmap of the differentially expressed genes (|log2 fold-change| > 1, and *P* < 0.05) in QBC939 cells. **L** The KEGG analyses were performed and top 20 enriched pathways among differentially expressed genes in (**K**) were illustrated by bubble plot. **M** QBC939 cells were prepared as described in (**A**–**J**) and western blot for protein expression of PKA, CREB, P-CREB in cAMP pathway. **N**, **O** QBC939 cells were prepared as described in (**A–J**) with or without the addition of H89, a PKA inhibitor. Flow cytometry to detect cell death (**N**) and intracellular lipid ROS (**O**). **A**–**J** and **M**–**O** The data are presented as the mean ± standard deviation from three independent experiments, *P < 0.05, **P < 0.01, ***P < 0.001, ****P < 0.0001.

Individual reports have recently shown that CYP1B1 is likely to affect tumor progression by regulating ferroptosis level [42, 43]. To determine the potential role of CYP1B1 in ferroptosis regulation in ECC cells, we first utilized a selective inhibitor of CYP1B1, TMS, to treat ECCs in combination with AKR1C1 inhibitor AKR1C1-IN-1. As expected, blockade of CYP1B1 activity led to a notable increase in cell proliferation and colony formation of AKR1C1-IN-1-treated ECC cells (Fig. 6A, B), and conversely a remarkable decrease in cell death, lipid peroxidation, and intracellular iron levels (Fig. 6C–E). Then, we used siRNA-mediated suppression of CYP1B1 in AKR1C1-depleted ECC cells, and similarly found that the effect of inducible AKR1C1 knockdown on promoting ferroptosis was significantly counteracted by silencing of CYP1B1 (Fig. 6F–J). Therefore, CYP1B1 mediates the ferroptosis-inducing effect of AKR1C1 depletion in ECC cells.

To further explore the underlying molecular mechanisms of how the AKR1C1–CYP1B1 axis modulates ferroptosis in ECC cells, we performed transcriptome sequencing in AKR1C1-depleted ECC cells with or without CYP1B1 siRNA. A total of 654 differentially expressed genes, including 107 upregulated genes and 553 downregulated genes, were identified (*P* value < 0.05 and |log_2_ fold-change| > 1) in ECC cells simultaneously treated with inducible AKR1C1 knockdown and CYP1B1 siRNA (Fig. 6K and Supplementary Fig. 2). KEGG pathway enrichment analysis revealed the top 20 significantly enriched pathways (Fig. 6L), of which, the cAMP signaling pathway ranked the first.

Recent studies have indicated that activation of the cAMP–PKA signaling pathway can promote ferroptosis in cardiomyocytes and liver cells [44, 45]. Therefore, we first analyzed changes in the expression of several important cAMP pathway-related proteins, including PKA, CREB, and p-CREB in ECC cells. As shown in Fig. 6M, inducible AKR1C1 knockdown upregulated the protein levels of PKA and p-CREB, thus gave rise to cAMP pathway activation. In contrast, further silencing of CYP1B1 with siRNA significantly rescued the accumulation of PKA and p-CREB protein levels in AKR1C1-depleted ECC cells. In line with these results, CYP1B1 inhibitor TMS counteracted the effect of AKR1C1 blockade on enhancing the protein levels of PKA and p-CREB. Furthermore, H89, a PKA inhibitor, was added into AKR1C1-depleted ECC cells in the presence or absence of CYP1B1 siRNA. Results indicated that PKA inhibitor attenuated the effect of AKR1C1 knockdown on promoting ferroptosis and lipid peroxidation in ECC cells (Fig. 6N, O). The above results reveal that AKR1C1 sensitizes ECC cells to ferroptosis induction via the CYP1B1–cAMP signaling axis.

### Depletion of endogenous AKR1C1 sensitizes cancer cells to ferroptosis in vivo and offers potent therapeutic strategy for ECC

**Fig. 7:**
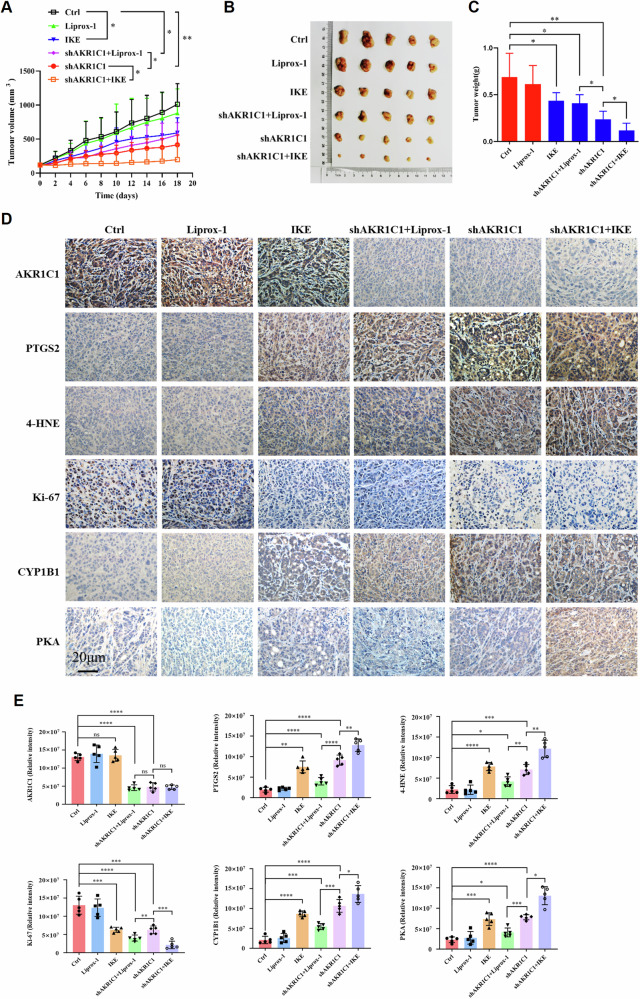
Depletion of endogenous AKR1C1 sensitizes cancer cells to ferroptosis in vivo and offers potent therapeutic strategy for ECC. **A**–**D** QBC939 cells harboring Dox-inducible shAKR1C1 were subcutaneously injected into nude mice and treated with normal feeding, DOX feeding, Liproxstatin-1 (i.p.), IKE (i.p.) or indicated combination (*n* = 5/group). **A** Tumor volumes of each treatment group were measured every 2 days. Data are presented as the mean ± standard, *n* = 5/group, *P < 0.05, **P < 0.01. **B** Images of resected tumors from each group were taken and **C** tumor weights were measured at day 18. Data are presented as the mean ± standard, *n* = 5/group, *P < 0.05, **P < 0.01. **D** Representative images of IHC staining for AKR1C1, PTGS2, 4-HNE, Ki67 (a well-known proliferation marker), CYP1B1 and PKA, all counter-stained with hematoxylin, were taken from sections of xenografted tumors from each group as indicated. **E** The mean staining intensity of AKR1C1, PTGS2, 4-HNE, Ki67, CYP1B1, and PKA in xenografted tumors. *** *P* < 0.001, **** *P* < 0.0001.

Finally, to elucidate the clinical relevance of the AKR1C1–CYP1B1 axis in ferroptosis regulation, we performed an in vivo xenograft experiment by subcutaneously inoculating nude mice with QBC939 ECC cells harboring Dox-inducible AKR1C1 knockdown. As shown in Fig. 7A–C, tumor growth decreased in mice treated with either imidazole ketone erastin (IKE) to induce ferroptosis or Dox diet to induce AKR1C1 knockdown, alone or in combination, in comparison with control mice. Notably, the combination of Dox feeding (shAKR1C1) with imidazole ketone erastin (IKE) exerted a more significant effect than either alone in suppression of tumor growth. As expected, ferroptosis inhibitor Liproxstatin-1 partially restored the suppression of tumor growth upon Dox feeding (shAKR1C1).

Immunohistochemical staining showed that Dox feeding-induced AKR1C1 knockdown significantly increased CYP1B1 and PKA expression in tumors, and combination therapy of Dox diet (shAKR1C1) and IKE further upregulated the expression of CYP1B1 and PKA. Moreover, the treatment with either Dox diet (shAKR1C1) or IKE–Dox combination led to an increase in the ferroptosis marker PTGS2 (a marker of oxidative stress and ferroptosis) and 4-hydroxynonenal (4-HNE, a lipid peroxidation product) in tumors (Fig. 7D, E). Overall, these results recapitulate the in vitro observations and suggest that AKR1C1 depletion could render cancer cells more sensitive to ferroptosis and the combination of targeting AKR1C1 with ferroptosis inducers might represent a promising strategy for ECC treatment.

## Discussion

With the development of high-throughput sequencing technology, genomic analysis has become a powerful tool to identify candidate targets for cancer therapy [46]. In this study, we conduct detailed genomic analysis of clinical tumor tissues from a cohort of 75 patients with ECC to identify candidate genes that might contribute to ECC pathogenesis, leading to the discovery of AKR1C1, a member of the AKR gene family, as a crucial player in ECC (Fig. 1). Clinically, AKR1C1 is highly expressed in human ECC tissues and closely correlated with tumor progression and poor prognosis (Fig. 2). Furthermore, we demonstrate that inducible AKR1C1 knockdown inhibits ECC tumor growth and triggers ECC cells to undergo ferroptosis (Figs. 3 and 4). Importantly, we uncover for the first time that AKR1C1 directly binds to the cytochrome P450 family member CYP1B1, a newly discovered mediator of ferroptosis. Mechanistically, AKR1C1 regulates CYP1B1 via two mechanisms. First, AKR1C1 degrades CYP1B1 protein stability in ubiquitin-proteasomal degradation-dependent manner. Second, AKR1C1 can decrease CYP1B1 mRNA level through transcriptional factor AHR (Fig. 5). We also provide evidence to show that under this specific context, the AKR1C1–CYP1B1 axis modulates ferroptosis of ECC cells by the cAMP–PKA signaling pathway (Fig. 6). Notably, in an ECC xenograft mouse model, AKR1C1 depletion sensitizes cancer cells to ferroptosis and synergizes with ferroptosis inducers to suppress tumor growth (Fig. 7).

AKR1C1 belongs to the AKR1C family, which has recently been defined to suppress ferroptosis by detoxicating the reactive molecules lipid peroxides [18]. Several studies have linked AKR1C1 to different types of cancer, including breast cancer, gastric cancer, prostate cancer, and cervical cancer, and regulates tumor cell proliferation, metastasis, and chemotherapy-resistance in previous studies [12–17]. In the present study, we provide the first evidence that AKR1C1 is upregulated in human ECC tissues, predicts poor prognosis and suppresses ferroptosis via the CYP1B1–cAMP signaling axis in ECC progression. These findings support an oncogenic role of AKR1C1 in ECC and suggest that AKR1C1 represents a potentially novel therapeutic target for ECC.

Cytochrome P450 (CYP) enzymes have been demonstrated as key players of ferroptosis [36–41]. However, there exist multiple CYP members and their potential roles in ferroptosis regulation are largely unknown. Cytochrome P450 1B1 (CYP1B1) is a member of the CYP superfamily and participates in metabolic events, including the metabolism of fatty acids. Interestingly, CYP1B1 is expressed in many extra-hepatic tissues and overexpressed in different types of tumors [47–49]. Although individual reports have recently shown that CYP1B1 is likely to affect tumor progression by regulating ferroptosis level [42, 43], the potential role of CYP1B1 in ferroptosis modulation in ECC cells remains unclear. In our study, we uncover for the first time that CYP1B1 mediates the ferroptosis-inducing effect of AKR1C1 depletion in ECC cells, and AKR1C1 functions to suppress ferroptosis in ECC cells by decreasing both the mRNA and protein stability of CYP1B1 (Figs. 5 and 6). However, the complete regulatory mechanism of the AKR1C1–CYP1B1 axis and its upstream signaling, especially in the process of ferroptotic cell death, needs further investigation.

Aberrations in cAMP–PKA signaling have been implicated across diverse human tumor types. Depending on the specific tumor context, cAMP signaling may exert either tumor-suppressive or tumor-promoting effects. Recent studies have also indicated that activation of the cAMP signaling pathway can promote ferroptosis in cardiomyocytes and liver cell lines [44, 50]. Here, our subsequent mechanistic analysis shows that the cAMP–PKA signaling pathway is the main pathway involved in the AKR1C1–CYP1B1 axis-mediated ferroptosis regulation in ECC cells (Figs. 6 and 7). To our knowledge, this is the first report documenting such findings.

Our findings also help to address a significant unmet clinical need. ECC is one of the cancer types with most unfavorable diagnosis due to its aggressive growth and early metastasis. Currently, there is no effective treatment for ECC other than complete resection. In this study, we provide in vitro and in vivo pre-clinical experiment data to show that the AKR1C1–CYP1B1–cAMP signaling axis is a promising therapeutic target for the treatment of ECC by ferroptosis induction. Strikingly, in vivo, AKR1C1 depletion renders cancer cells more sensitive to ferroptosis and synergizes with ferroptosis inducers to suppress tumor growth much more potently than either treatment alone (Fig. 7). Additionally, our data also support the rationale of AKR1CI as a novel biomarker for ECC diagnosis and prognosis.

In conclusion, we identify AKR1C1 as a crucial player in ECC, which functions to downregulate both mRNA and protein stability of CYP1B1 to suppress ferroptosis. Our finding that AKR1C1 depletion can promote ECC ferroptosis in both cells and xenograft models via the CYP1B1–cAMP signaling axis, provide biological rationale for targeting AKR1C1, especially in combination with ferroptosis inducers, as a new promising therapeutic strategy for the treatment of ECC.

## Materials and methods

### Human tissue samples

Two sets of human extrahepatic cholangiocarcinoma (ECC) tissue samples were obtained from newly diagnosed ECC patients without radiotherapy, chemotherapy, or immunotherapy prior to the surgery at Shanghai Eastern Hepatobiliary Surgery Hospital (Naval Medical University, Shanghai, China). Set 1 contained 23 pairs of ECC tissues and adjacent non-tumor tissues. Set 2 contains samples from 55 cases of ECC patients and matched adjacent tissues with survival follow-up information for 5 years. The characteristics of this cohort of 55 patients with ECC are listed in Table 1. All specimens were confirmed by histopathological examination and immediately frozen in liquid nitrogen. Tumor stages were histologically classified according to the 2010 American Joint Cancer Committee Tumor-Node-Metastasis (TNM) classification (stages I–IV). Tumor differentiation degrees were defined according to the World Health Organization criteria (well differentiated, moderately differentiated and poorly differentiated). A total of 70 paraffin-embedded various solid tumor tissues and paired adjacent normal tissue samples, including 10 matched intrahepatic cholangiocarcinoma, 10 matched ECC, 10 matched hepatocellular carcinoma, 10 matched gastric cancer, 10 matched pancreatic cancer, 10 matched colorectal cancer and 10 matched esophageal cancer, were also collected from Shanghai Eastern Hepatobiliary Surgery Hospital. The Institutional Research Ethics Committee of Shanghai Eastern Hepatobiliary Surgery Hospital approved the study protocols and the informed consent of each patient was required (approval number: EHBHKY 2016-01-009, EHBHKY2018-02-014).

### RNA sequencing (RNA-seq) and data analysis

High-throughput RNA sequencing and data analysis were conducted by Shanghai OE BIOTECH Co., LTD. (Shanghai, China). Briefly, total RNA was extracted with TRIzol reagent (Thermo Fisher Scientific, Waltham, MA, USA) following the manufacturer’s protocol. Extracted RNA samples were quantified using NanoDrop 2000 spectrophotometer (Thermo Fisher Scientific, Waltham, MA, USA) and RNA integrity was checked using Agilent 2100 Bioanalyzer (Agilent Technologies, Santa Clara, CA, USA). For RNA sequencing, strand-specific RNA-seq libraries were prepared using TruSeq Stranded mRNA LT Sample Prep Kit (Illumina, San Diego, CA, USA), subjected to quality control using a Bioanalyzer 2100 (Agilent, Santa Clara, CA, USA) and were sequenced using a NovaSeq 6000 platform (Illumina, San Diego, CA, USA). Quality control (QC) of raw reads from all samples was performed using Trimmomatic software. Spliced transalignment to a reference (HISAT2) software was used to perform sequence alignment on the clean reads of each sample. Differential expression analysis was performed using the DESeq (2012) R package. Hierarchical cluster analysis of differentially expressed genes (DEGs) was performed to demonstrate the expression pattern of genes in different groups and samples. GO enrichment and KEGG pathway enrichment analysis of DEGs were performed respectively using R based on the hypergeometric distribution.

### Cell lines and cell culture

The human extrahepatic CCA cell line QBC939, intrahepatic CCA cell line HCCC9810, and hepatoma cell line Huh7 were provided by WY (Shanghai Eastern Hepatobiliary Surgery Hospital, China). Human esophageal cancer cell line TE-1, pancreatic cancer cell line PANC1, gastric carcinoma cell line MGC823, and colon carcinoma cell HCT8 were obtained from Shanghai Cell Bank (Shanghai Biological Sciences, Chinese Academy of Sciences, Shanghai, China). All cell lines were cultured in RPMI 1640 medium (Gibco, Grand Island, NY, USA) or Dulbecco’s Modified Eagle Medium (DMEM) (Gibco, Grand Island, NY, USA) supplemented with 10% heat-inactivated fetal bovine serum (FBS) (Gibco, Grand Island, NY, USA), 100 U/mL of penicillin, and 100 μg/mL of streptomycin (Thermo Fisher Scientific, Waltham, MA, USA) at 37 °C in humidified air containing 5% carbon dioxide (CO_2_). The cell lines used in this study have been authenticated.

### Quantitative RT-PCR (qRT-PCR)

Total RNA used for qRT-PCR was purified by RNAiso Plus (Takara-Bio, Dalian, China) following the manufacturer’s protocol. For qRT-PCR, cDNAs were synthesized with the PrimeScript RT reagent kit (Takara-Bio, Dalian, China) and PCR reactions were performed with SYBR Premix EX Taq TM (Takara-Bio, Dalian, China). Gene expression was normalized to the control gene glyceraldehyde-3-phosphate dehydrogenase (GAPDH) to calculate relative expression changes. The primer sequences for qRT-PCR were listed as follows:

AKR1C1-Forward, 5′-GGCTTTGTTAGGCAACTGTGT-3′ and AKR1C1-Reverse, 5′-AAGGCAGCGAAGGATTCAGA-3′;

CYP1B1-Forward, 5′-AAGTTCTTGAGGCACTGCGA-3′ and CYP1B1-Reverse, 5′- CAGTGATAGTGGCCGGTACG-3′;

GAPDH-Forward, 5′-CTTAGCACCCCTGGCCAAG-3′ and GAPDH-Reverse, 5′-TGGTCATGAGTCCTTCCACG-3′.

### Antibodies and reagents

The following antibodies were used for immunoblot or immunohistochemistry analysis: AKR1C1 (R&D, Minnesota, USA), CYP1B1 (ProteinTech Group, Chicago, IL, USA), PKA (CST, Beverly, MA, USA), CREB (CST, Beverly, MA, USA), P-CREB (CST, Beverly, MA, USA), Beta tubulin (CST, Beverly, MA, USA), horseradish peroxidase (HRP)-conjugated anti-mouse secondary antibody (CST, Beverly, MA, USA), horseradish peroxidase (HRP)-conjugated anti-rabbit secondary antibody (CST, Beverly, MA, USA), AKR1C1 (Abcam, Cambridge, MA, USA), PTGS2 (CST, Beverly, MA, USA), 4-HNE (R&D, Minnesota, USA), and Ki67 (CST, Beverly, MA, USA). The following chemicals were commercially obtained: doxycycline (Dox) and puromycin from Selleck Chemicals (Houston, TX, USA); Ferrostatin-1, Z-VAD-FMK, GSK′872, erastin, RSL3, Liproxstatin-1 and IKE from MedChemExpress (Monmouth Junction, NJ).

### Western blot analysis

Briefly, after collecting protein lysates in 2% SDS, we measured the protein concentration of each sample and separated the samples on a 7.5%–12.5% SDS–PAGE gel (Bio-Rad Laboratories Inc., Hercules, CA, USA). Next, the proteins were transferred to a membrane, the membrane was incubated with the appropriate antibodies overnight at 4 °C, followed by incubation with secondary antibodies. Immunodetection was visualized using the enhanced chemiluminescence system (ECL, Thermo Fisher Scientific, Waltham, MA, USA) according to the manufacturer’s instructions.

### Immunohistochemistry (IHC) assay

For paraffin-embedded tissue sections, deparaffinization, rehydration, antigen retrieval, and endogenous peroxidase inactivation (3% hydrogen peroxide) were performed. After blocking, tumor sections were incubated with HRP-conjugated primary antibodies at 4 °C overnight. Staining was performed using the HRP-IHC kit (Maixin Fuzhou, China) according to the manufacturer’s instructions. The AKR1C1 expression of human ECC tissues was interpreted independently by two pathologists blinded. In immunohistochemical analysis, the expression of the target protein is evaluated based on the location and intensity of positive staining in the tissue: Score 0, positive staining in 0%–5% of cells; Score 1, weak positive staining in 6%–35% of cells; Score 2, moderate positive staining in 36%–55% of cells. Score 3, strong positive staining in 56%–100% of cells. Each relevant protein level is assessed individually, and the average score for staining of each protein is reported.

### Generation of inducible AKR1C1 knockdown or overexpression cells

The lentiviral doxycycline (Dox)-inducible AKR1C1 shRNA or overexpression system was conducted by Genechem (Shanghai, China). Briefly, shRNA for AKR1C1 (targeting sequence: 5′-CACCAAATTGGCAATTGAA-3′) was cloned into the Dox-inducible lentiviral vector pLenti-Tet-MCS-Puro (GeneChem, Shanghai, China) to generate pTet-shAKR1C1 plasmid. The coding sequence of AKR1C1 was obtained via PCR amplification and then subcloned into the Dox-inducible lentiviral vector pLenti-Tet-MCS-Puro to generate pTet-ovAKR1C1 plasmid. Lentiviral particles were produced by co-transfection of pTet-shAKR1C1 or pTet-ovAKR1C1 plasmid with the packaging plasmids into HEK293T packaging cells following the lentivirus packaging protocol of GeneChem. Media was changed 6 h after transfection, and the virus-containing supernatant was collected and filtered 48 h after transfection. QBC939 cells in 6-well tissue culture plates were infected with lentiviral particles containing pTet-shAKR1C1 or pTet-ovAKR1C1 plasmid and subject to puromycin (4 µg/ml) selection for at least 7 days to obtain stable AKR1C1 knockdown or overexpression cells. Then Dox (5 µg/ml) was added to the culture media for 3 days.

### RNA interference

siRNAs were commercially synthesized (Genomeditech, Shanghai, China) and transfected into cells using Lipofectamine RNAiMAX (Thermo Fisher Scientific, Waltham, MA, USA). siRNA sequences targeting human genes were as follows:

si-CYP1B1, 5′-GCAUGAUGCGCAACUUCUU-3′.

### Cell Counting Kit-8 (CCK-8) assay

QBC939 cells containing Dox-inducible shAKR1C1 were split into 96-well plates (6 × 10^3^ cells, 100 µL/well, three replicates) and incubated with or without Dox (5 µg/ml) for 0, 12, 24, 36, 48, 60, and 72 h at 37 °C. CCK-8 solution (Vazyme Biotech Co., Nanjing, China) was added to each well for 2 h, and then absorbance at 450 nm was measured.

### Colony formation assay

QBC939 cells were seeded at an appropriate density in a 6-well plate and cultured overnight at 37 °C in humidified air containing 5% CO_2_. Upon stable adhesion and growth, the culture medium was changed every 2 days. After 14 days of cell cultivation, the medium was aspirated, and cells were washed with PBS. The cells were fixed with 4% paraformaldehyde (Beyotime Biotechnology, Shanghai, China) for 10 min, followed by staining of cell colonies with crystal violet (Sangon, Shanghai, China). Subsequently, photographic documentation and colony counting were performed.

### Transfection

The full-length human AKR1C1 (NM_ 001353.6) cDNA was cloned into the pcDNA3.1 plasmid (Invitrogen, Waltham, MA, USA) and verified by sequencing. ECC cells were cultured in 12-well plates at a concentration of 1 × 10^5^/well. These pcDNA or siRNAs were transfected into cells by Lipofectamine 3000 reagent (Invitrogen, Waltham, MA, USA) according to the manufacturer’s instructions. Flow assay, RT-PCR, or western blot analyses were conducted 48 h post-transfection.

### Flow cytometry for cell death and lipid peroxidation analysis

Cells were stained with 3 nM SYTOX GREEN DEAD CELL STAIN (Thermo Fisher Scientific, Waltham, MA, USA) to monitor cell death using an FC 500 flow cytometer (Beckman Coulter, Brea, CA, USA). Percentage of cell death was calculated as SYTOX GREEN^+^ cell number over total cell number. To analyze lipid peroxidation, cells were stained with 5 µM BODIPY C11 (Thermo Fisher Scientific, Waltham, MA, USA) for 30 min after indicated treatment. Labeled cells were trypsinized, resuspended in PBS plus 2% FBS, and then subjected to flow cytometry analysis.

### Iron assay

Cellular Fe^2+^ and total iron content were determined using the Sigma-Aldrich Iron Assay Kit. Cells were homogenized in 400 µL of buffer and centrifuged at 4 °C, 16,000 × *g* for 10 min. Then 50 µL of sample was taken into a 96-well plate and an additional 50 µL of assay buffer was added to each well. For Fe^2+^ measurement, 5 µL of iron assay buffer was added to each sample. For total iron measurement, 5 µL of iron reducer was introduced to each well. The 96-well plate was incubated in the dark at room temperature for 30 min. Subsequently, 100 µL of iron probe was added to each sample, followed by incubation in the dark at room temperature for 1 h. Absorbance was measured at 593 nm.

### Immunofluorescence assay

QBC939 cells were seeded at an appropriate density in a 24-well plate. After 24 h, cells were fixed with 4% paraformaldehyde for 15 min, followed by permeabilization with 0.5% Triton X-100 for 10 min. Subsequently, non-specific binding was blocked with PBS containing 5% goat serum. The cells were then incubated overnight at 4 °C with the primary antibody. After washing, cells were incubated with the secondary antibody for 1 h in the dark at room temperature. For nuclear staining, DAPI (Beyotime Biotechnology, Shanghai, China) was applied for 10 min at room temperature. The stained cells were visualized using a confocal microscope (Leica, Germany).

### Co-immunoprecipitation (Co-IP)

In order to immunoprecipitate exogenously expressed or endogenous proteins, cellular lysis was carried out using NP-40 lysis buffer containing a proteinase inhibitor. The cells were then incubated overnight at 4 °C with either the primary antibody specific to the target protein or an IgG control in a rotating culture chamber. Subsequently, the immunocomplexes were incubated with 10 μl of Protein A/G magnetic beads (Beyotime Biotechnology, Shanghai, China) for 1 h at 4 °C. The immunoprecipitates were subjected to triple washes with PBST buffer, followed by immunoblot analysis.

### In vivo xenograft mouse model

Five- to six-week-old male BALB/c nude mice (Jh-labanimal, Shanghai, China) were housed and monitored in the Animal Research Facility at Naval Medical University. All experimental procedures and protocols were approved by the Animal Care and Use Committee of Naval Medical University (approval number: 2021SLYS04). Mice were injected in the right flank with 5 × 10^6^ Dox-inducible shAKR1C1 QBC939 cells suspended in 0.1 ml PBS. Tumors were measured with callipers every 2 days. When tumors reached a mean volume of 100 mm^3^, mice with similarly sized tumors were grouped into four treatment groups (6 mice for each group). For control group, mice were given intraperitoneal (i.p.) injections of 0.9% sterile saline for 2 days. At the same time, mice were provided with daily normal diet. For Liproxstatin-1 group, mice were treated with Liproxstatin-1 (10 mg/kg body weight) via daily i.p. injection and provided with daily normal diet. For IKE group, mice were treated with IKE (50 mg/kg body weight) via daily i.p. injection and provided with daily normal diet. For Dox group, mice were given i.p. injections of Doxycycline (50 mg/kg body weight) for 2 days. At the same time, mice were provided with daily Dox (4 mg/kg body weight) diet. For combination of DOX and Liproxstatin-1 group, mice were given i.p. injections of Doxycycline (50 mg/kg body weight) for 2 days. At the same time, mice were treated with Liproxstatin-1 (10 mg/kg body weight) via daily i.p. injection and provided with daily Dox (4 mg/kg body weight) diet. For combination of DOX and IKE group, mice were given i.p. injections of Doxycycline (50 mg/kg body weight) for 2 days. At the same time, mice were treated with IKE (50 mg/kg body weight) via daily i.p. injection and provided with daily Dox (4 mg/kg body weight) diet. For all experiments, mice were sacrificed at a pre-determined endpoint. If any tumor exceeded a volume of 2000 mm^3^, 1.5 cm in diameter, or 10% of body weight, the mice would immediately be euthanized. At the end of the study, mice were euthanized with CO_2_ and tumors were taken for measurement of weight, followed by immunohistochemical staining.

### Quantitative analysis

Statistical analysis of immunohistochemical staining of xenograft tumor sections. Five randomly selected fields of view in each section were photographed under the microscope, and the mean value was used as a reliable indicator for quantitative analysis of the immunohistochemical results. The analysis was conducted using Image Pro Plus 7.0 image analysis software. The positive research cell area (Area), average optical density value (Mean Density, MD) value, and integral optical density (IOD) value were determined by the formula: IOD = Area × MD.

### Statistical analysis

All statistical analyses were performed using GraphPad Prism 8.0 or SPSS 21.0 software. Data are presented as mean ± SD from three independent experiments. *P* values are calculated by Student’s *t*-test or one-way ANOVA as indicated in figure legends. Survival curves were analyzed using the Kaplan–Meier method and the log-rank test. *P* values < 0.05 were considered statistically significant for all experiments.

## Supplementary information

Original western blots

Supplementary Figures

## Data Availability

All data generated or analyzed during this study are included in the article and its Supplementary Information files or are available from the corresponding author upon reasonable request.
