# Supplementary material for: The AKR1C1–CYP1B1–cAMP signaling axis controls tumorigenicity and ferroptosis susceptibility of extrahepatic cholangiocarcinoma
Source: Cell Death Differ. 2024 Oct 30;32(3):506–20. doi: 10.1038/s41418-024-01407-1 (PMC11894074; doi:10.1038/s41418-024-01407-1)
Supplement: Supplementary file 1 — Original western blots [file 41418_2024_1407_MOESM1_ESM.pptx]

## Slide 1
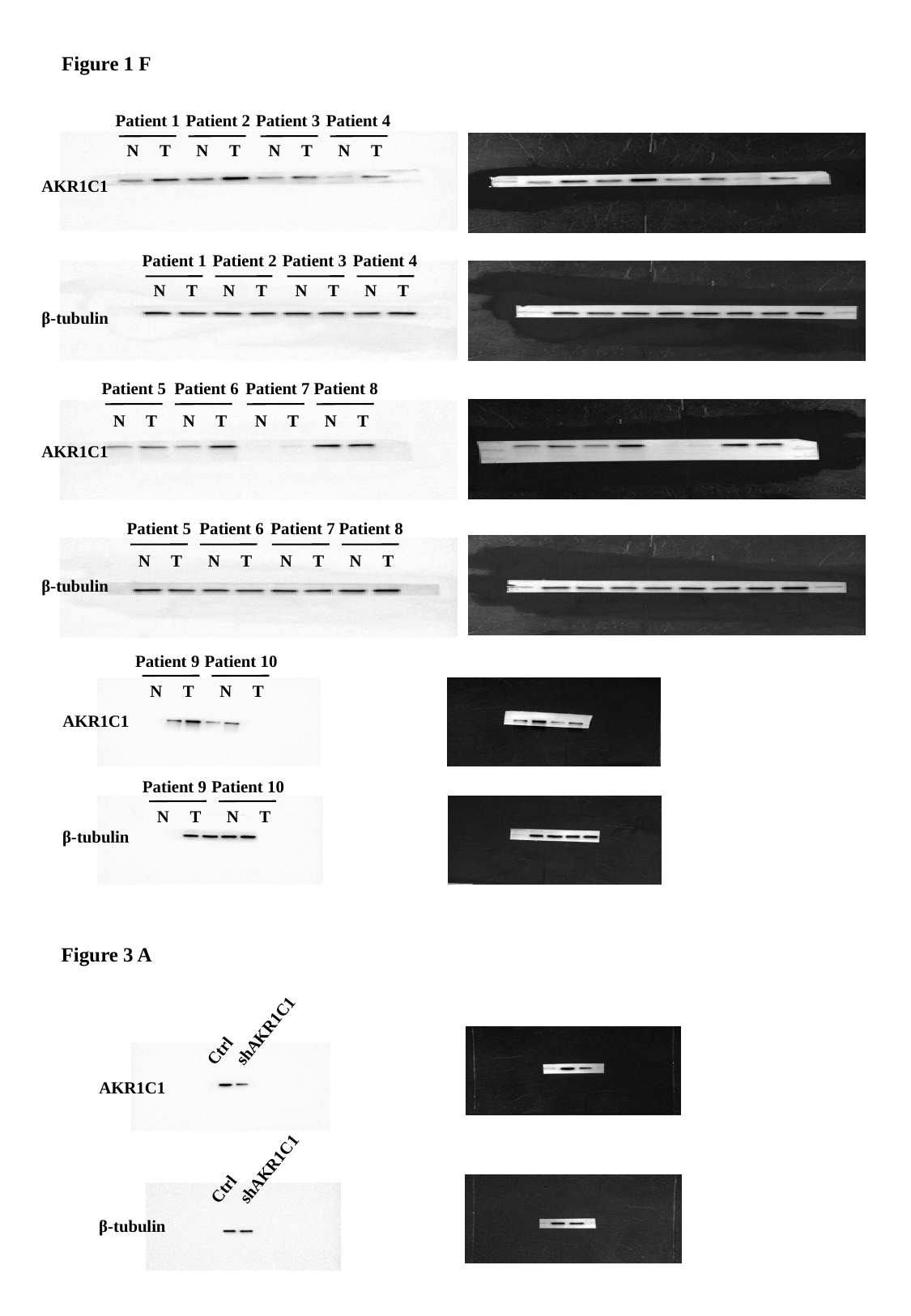

Figure 1 F
Patient 1
Patient 2
Patient 3
Patient 4
N
T
N
T
N
T
N
T
AKR1C1
Patient 1
Patient 2
Patient 3
Patient 4
N
T
N
T
N
T
N
T
β-tubulin
Patient 5
Patient 6
Patient 7
Patient 8
N
T
N
T
N
T
N
T
AKR1C1
Patient 5
Patient 6
Patient 7
Patient 8
N
T
N
T
N
T
N
T
β-tubulin
Patient 9
Patient 10
N
T
N
T
AKR1C1
Patient 9
Patient 10
N
T
N
T
β-tubulin
Figure 3 A
shAKR1C1
Ctrl
AKR1C1
shAKR1C1
Ctrl
β-tubulin

## Slide 2
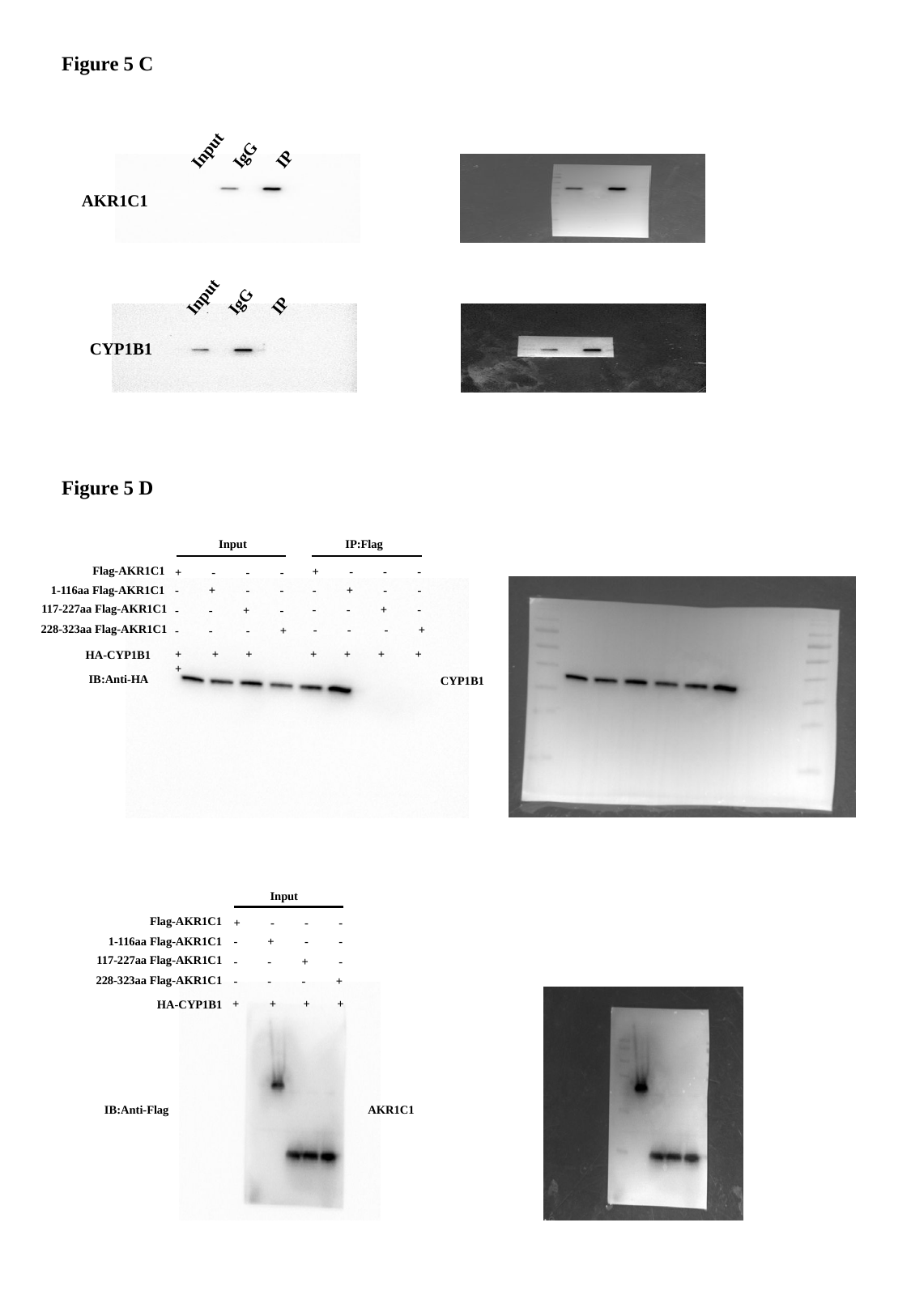

Figure 5 C
Input
IP
IgG
AKR1C1
Input
IP
IgG
CYP1B1
Figure 5 D
IP:Flag
Input
Flag-AKR1C1
+ - - -
+ - - -
- + - -
1-116aa Flag-AKR1C1
- + - -
117-227aa Flag-AKR1C1
- - + -
- - + -
228-323aa Flag-AKR1C1
- - - +
- - - +
+ + + +
+ + + +
HA-CYP1B1
IB:Anti-HA
CYP1B1
Input
Flag-AKR1C1
+ - - -
1-116aa Flag-AKR1C1
- + - -
117-227aa Flag-AKR1C1
- - + -
228-323aa Flag-AKR1C1
- - - +
+ + + +
HA-CYP1B1
IB:Anti-Flag
AKR1C1

## Slide 3
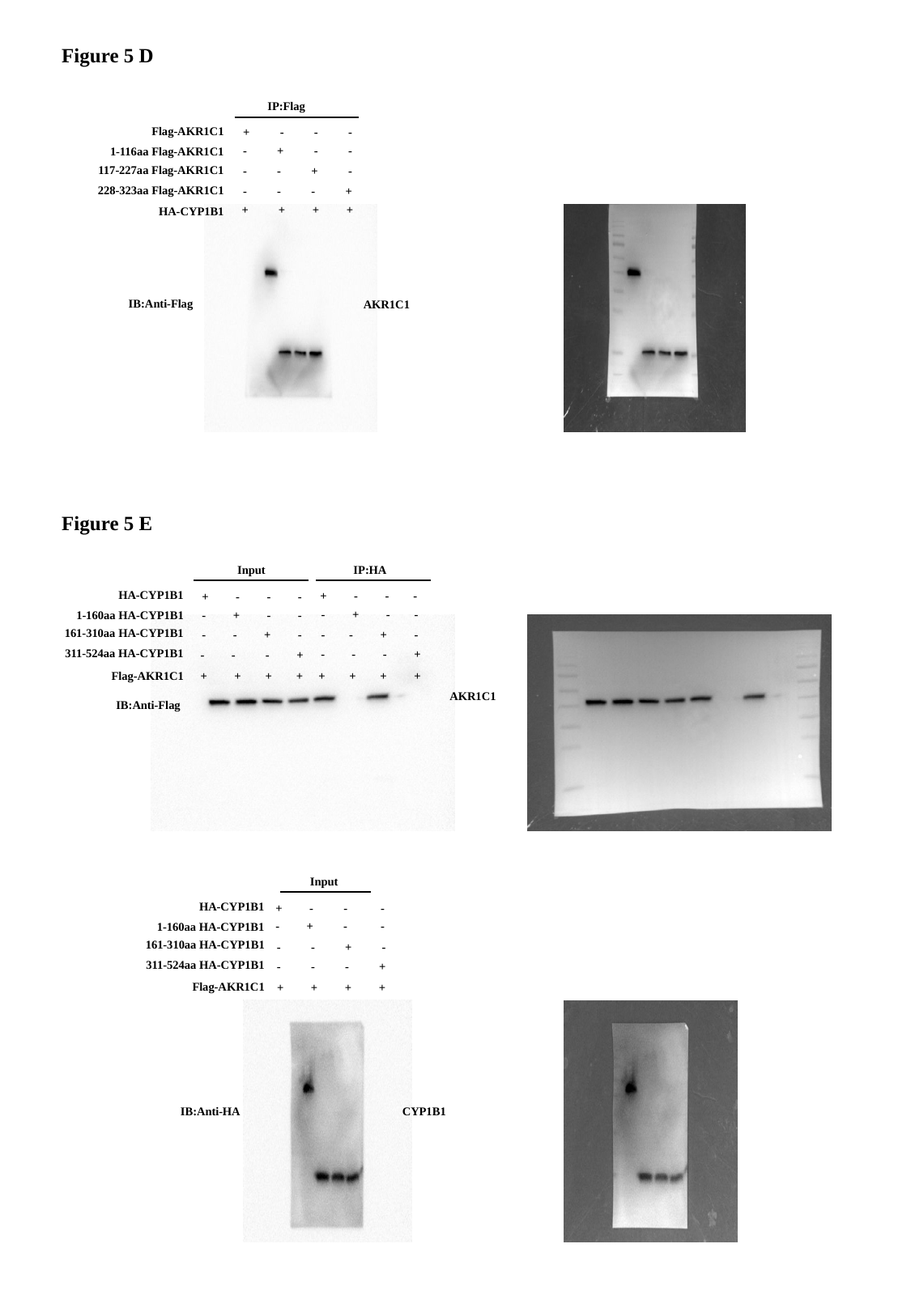

Figure 5 D
IP:Flag
Flag-AKR1C1
+ - - -
- + - -
1-116aa Flag-AKR1C1
117-227aa Flag-AKR1C1
- - + -
228-323aa Flag-AKR1C1
- - - +
+ + + +
HA-CYP1B1
IB:Anti-Flag
AKR1C1
Figure 5 E
Input
IP:HA
HA-CYP1B1
+ - - -
+ - - -
- + - -
- + - -
1-160aa HA-CYP1B1
161-310aa HA-CYP1B1
- - + -
- - + -
311-524aa HA-CYP1B1
- - - +
- - - +
Flag-AKR1C1
+ + + +
+ + + +
AKR1C1
IB:Anti-Flag
Input
HA-CYP1B1
+ - - -
- + - -
1-160aa HA-CYP1B1
161-310aa HA-CYP1B1
- - + -
311-524aa HA-CYP1B1
- - - +
Flag-AKR1C1
+ + + +
IB:Anti-HA
CYP1B1

## Slide 4
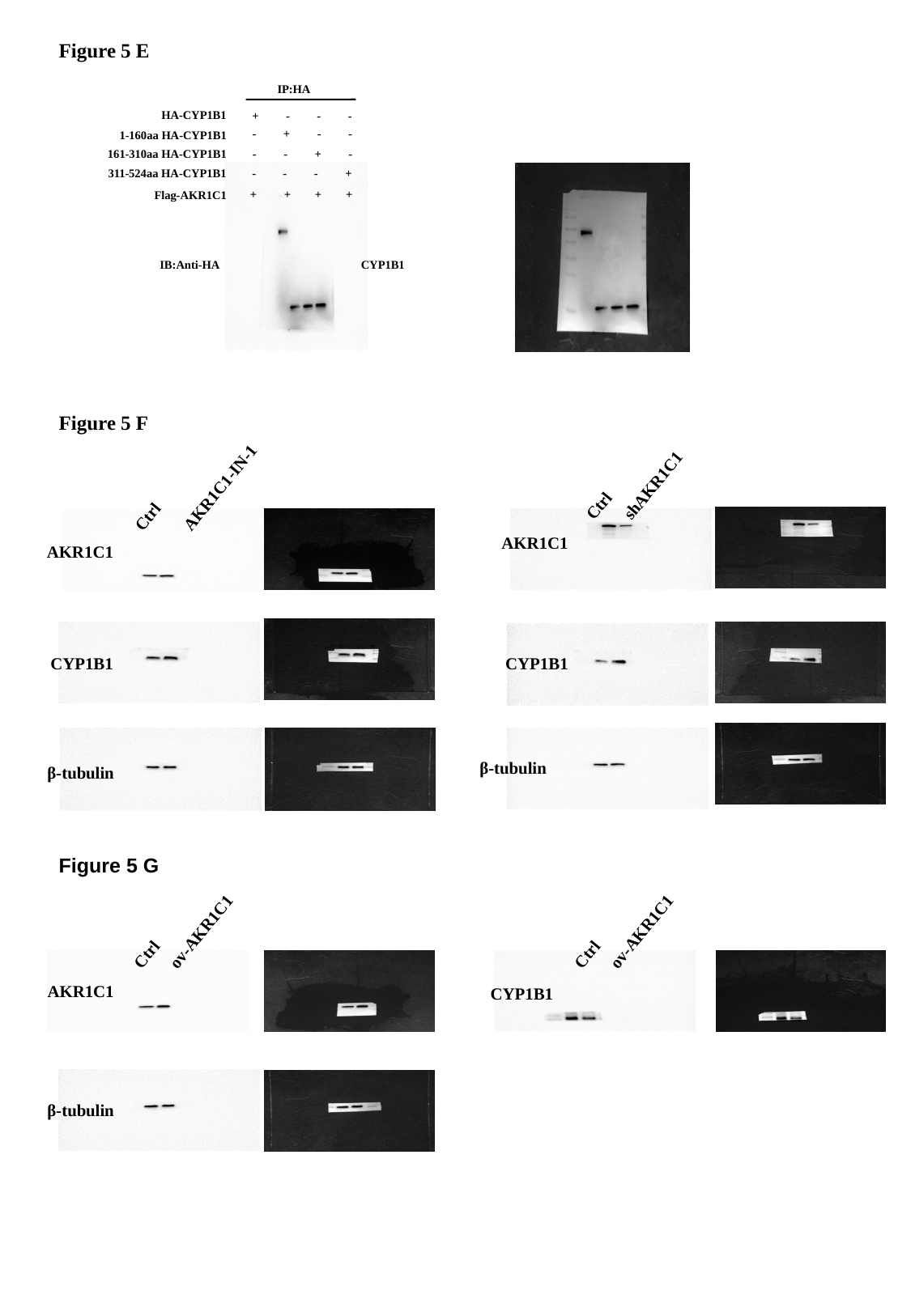

Figure 5 E
IP:HA
HA-CYP1B1
+ - - -
- + - -
1-160aa HA-CYP1B1
- - + -
161-310aa HA-CYP1B1
- - - +
311-524aa HA-CYP1B1
+ + + +
Flag-AKR1C1
IB:Anti-HA
CYP1B1
Figure 5 F
AKR1C1-IN-1
shAKR1C1
Ctrl
Ctrl
AKR1C1
AKR1C1
CYP1B1
CYP1B1
β-tubulin
β-tubulin
Figure 5 G
ov-AKR1C1
ov-AKR1C1
Ctrl
Ctrl
AKR1C1
CYP1B1
β-tubulin

## Slide 5
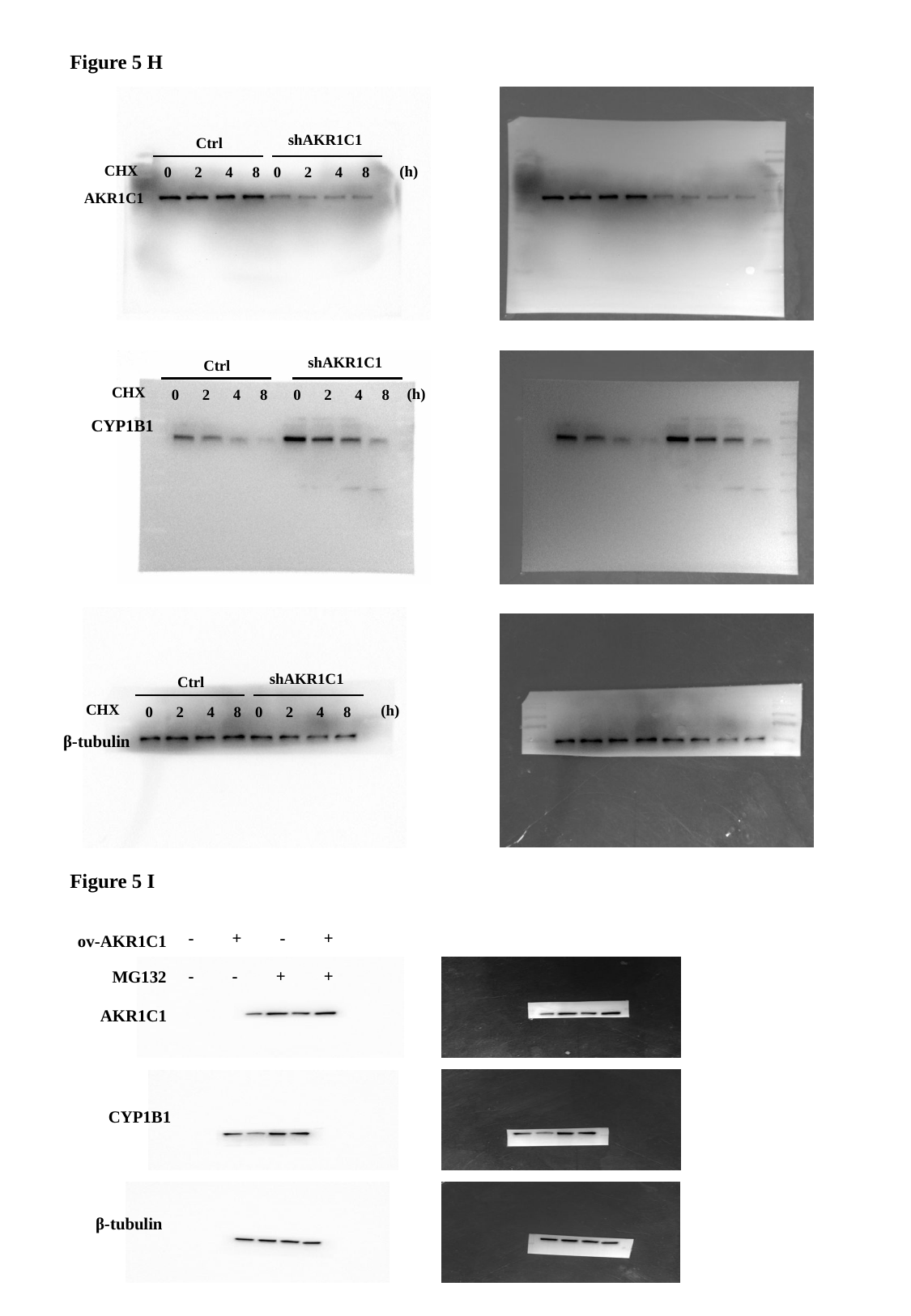

Figure 5 H
shAKR1C1
Ctrl
CHX
(h)
0 2 4 8
0 2 4 8
AKR1C1
shAKR1C1
Ctrl
CHX
(h)
0 2 4 8
0 2 4 8
CYP1B1
shAKR1C1
Ctrl
CHX
(h)
0 2 4 8
0 2 4 8
β-tubulin
Figure 5 I
- + - +
ov-AKR1C1
- - + +
MG132
AKR1C1
CYP1B1
β-tubulin

## Slide 6
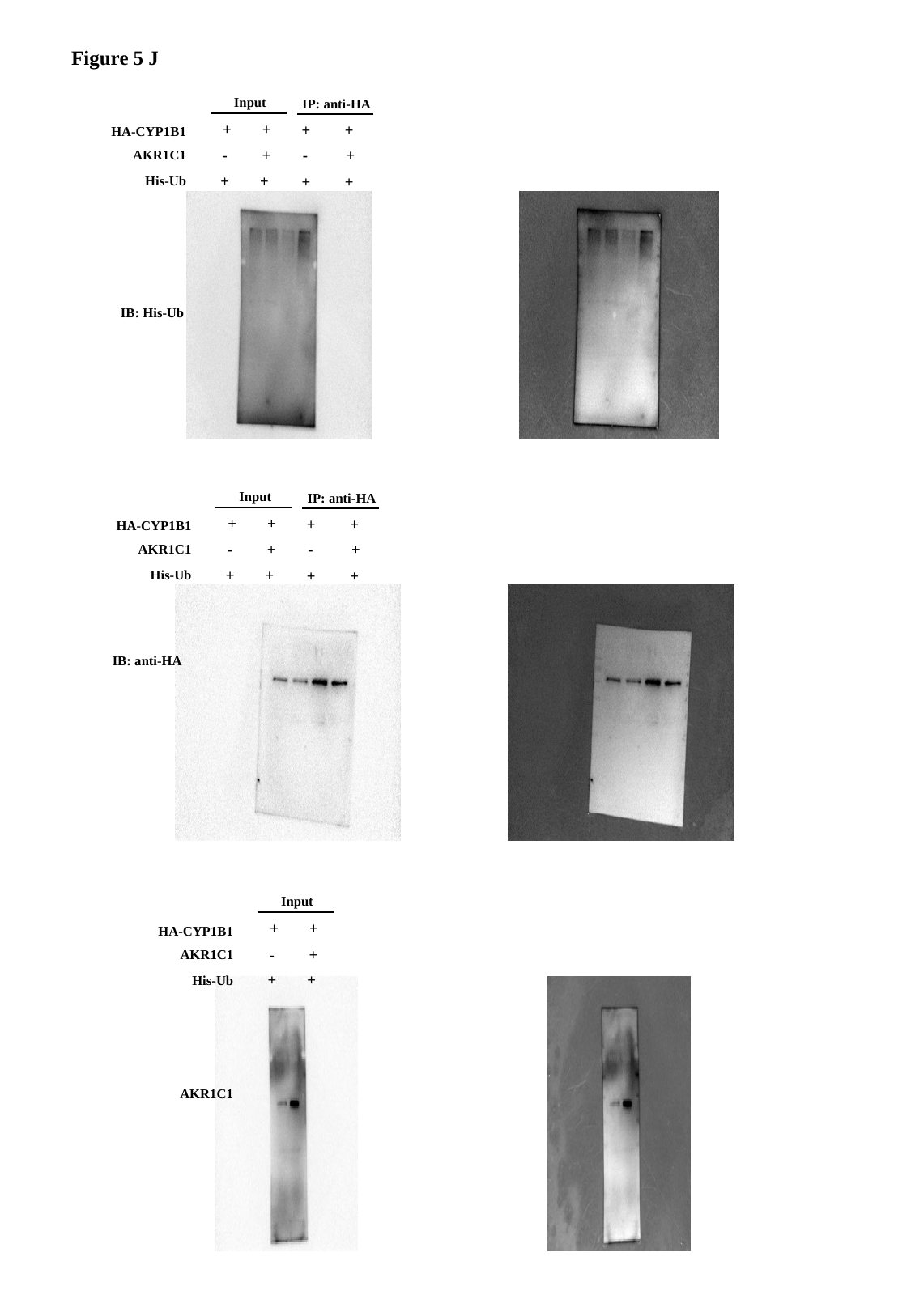

Figure 5 J
Input
IP: anti-HA
 + +
 + +
HA-CYP1B1
 - +
 - +
AKR1C1
 + +
 + +
His-Ub
IB: His-Ub
Input
IP: anti-HA
 + +
 + +
HA-CYP1B1
 - +
 - +
AKR1C1
 + +
 + +
His-Ub
IB: anti-HA
Input
 + +
HA-CYP1B1
 - +
AKR1C1
 + +
His-Ub
AKR1C1

## Slide 7
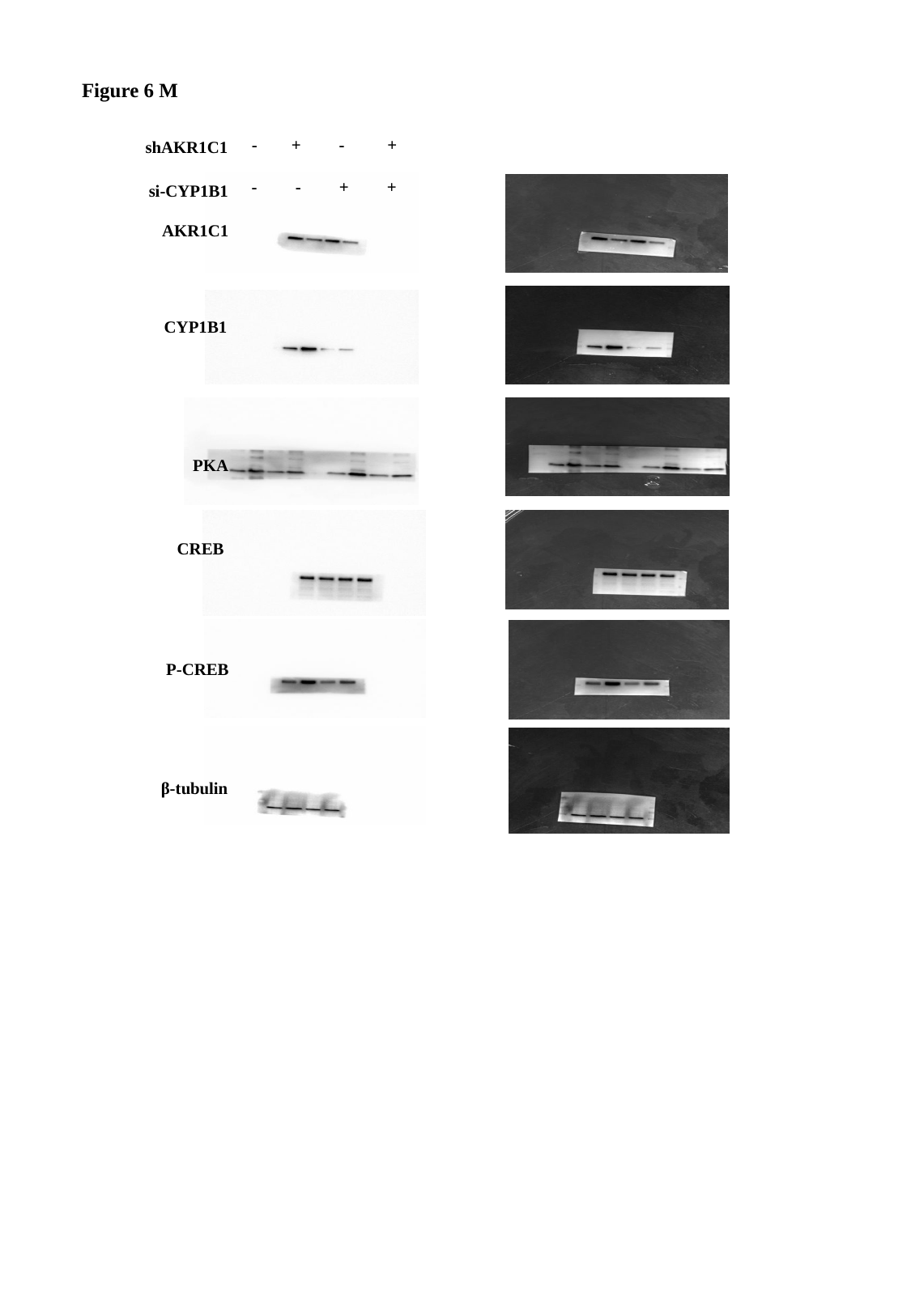

Figure 6 M
- + - +
shAKR1C1
- - + +
si-CYP1B1
AKR1C1
CYP1B1
PKA
CREB
P-CREB
β-tubulin

## Slide 8
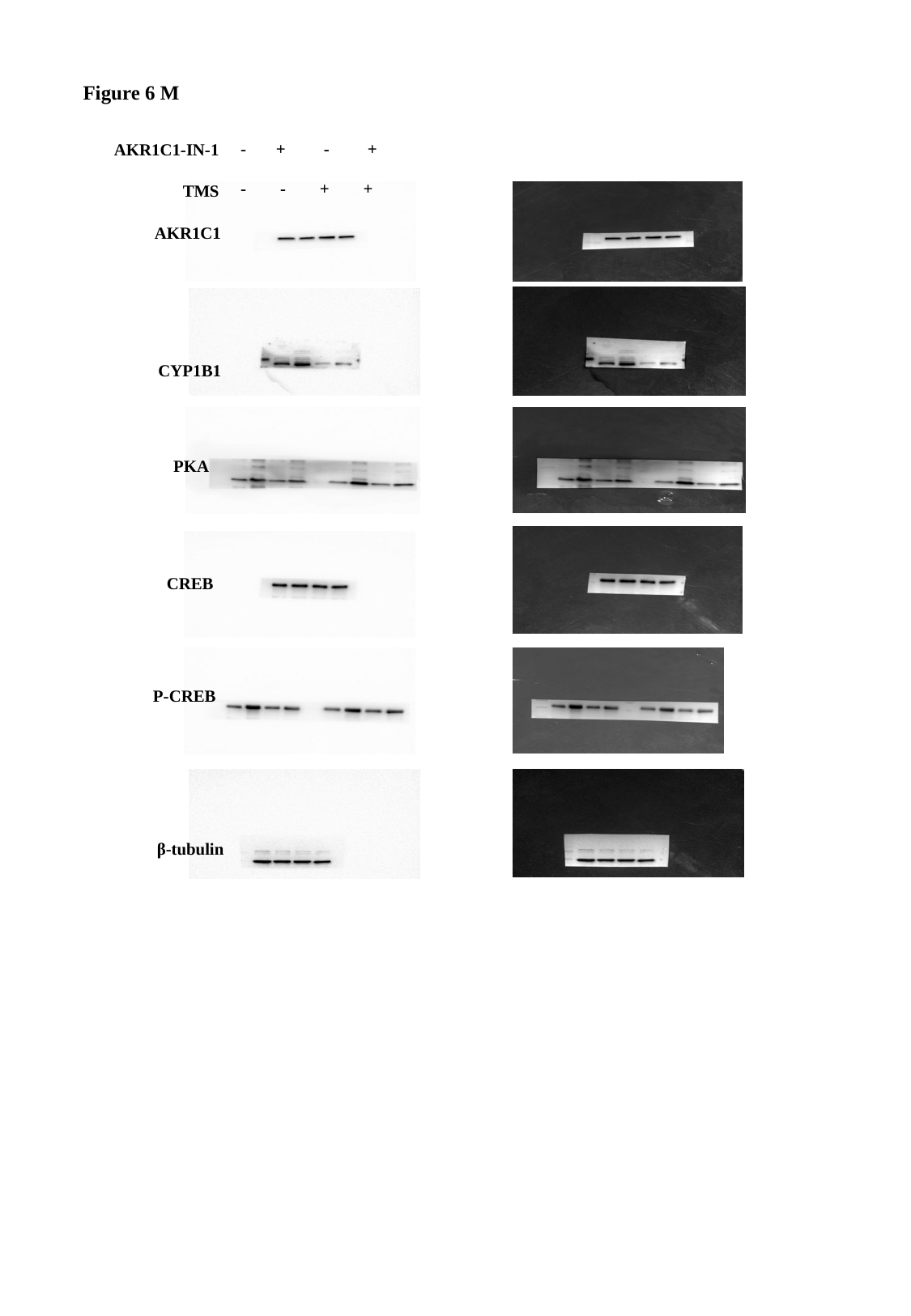

Figure 6 M
- + - +
AKR1C1-IN-1
- - + +
TMS
AKR1C1
CYP1B1
PKA
CREB
P-CREB
β-tubulin
