# Supplementary material for: The AKR1C1–CYP1B1–cAMP signaling axis controls tumorigenicity and ferroptosis susceptibility of extrahepatic cholangiocarcinoma
Source: Cell Death Differ. 2024 Oct 30;32(3):506–20. doi: 10.1038/s41418-024-01407-1 (PMC11894074; doi:10.1038/s41418-024-01407-1)
Supplement: Supplementary file 2 — Supplementary Figures [file 41418_2024_1407_MOESM2_ESM.docx]

**Supplementary**


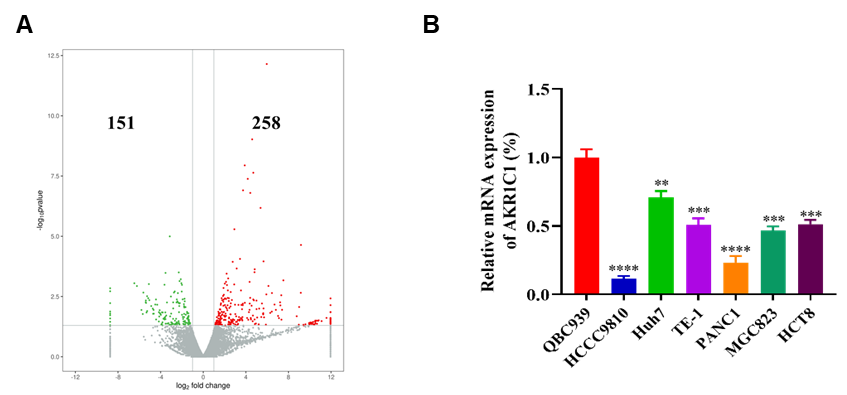


**Supplementary Figure 1.** RT-PCR analysis of AKR1C1 expression in extrahepatic CCA cell line (QBC939), intrahepatic CCA cell line (HCCC9810), hepatoma cell line (Huh7), esophageal cancer cell line (TE-1), pancreatic cancer cell line (PANC1), gastric carcinoma cell line (MGC823), and colon carcinoma cell (HCT8). Data are presented as the mean ± standard deviation from 3 independent experiments, ** P <0.01, *** P <0.001, **** P <0.0001.


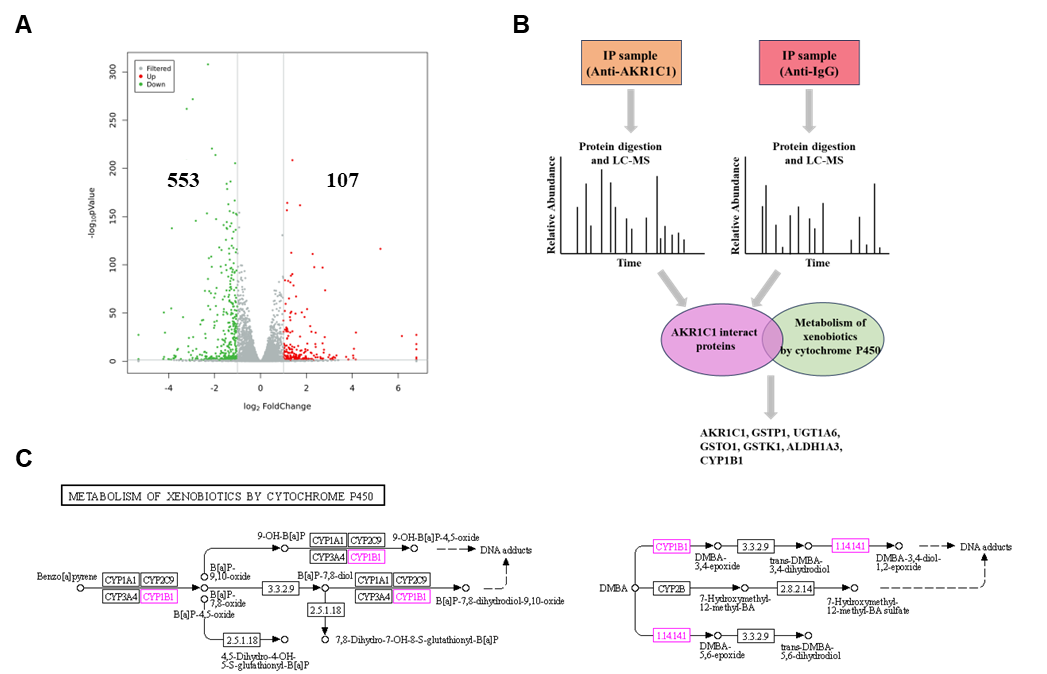


**Supplementary Figure 2.** **(A)** Volcano plot of the differentially expressed genes in Figure 6K. **(B)** A work chart showing the strategy to identify proteins interacting with AKR1C1. **(C)** The KEGG pathway map of Metabolism of xenobiotics by cytochrome P450.
